# Potential Applications of NRF2 Inhibitors in Cancer Therapy

**DOI:** 10.1155/2019/8592348

**Published:** 2019-04-11

**Authors:** Emiliano Panieri, Luciano Saso

**Affiliations:** Department of Physiology and Pharmacology “Vittorio Erspamer”, Sapienza University, P.le Aldo Moro 5, 00185 Rome, Italy

## Abstract

The NRF2/KEAP1 pathway represents one of the most important cell defense mechanisms against exogenous or endogenous stressors. Indeed, by increasing the expression of several cytoprotective genes, the transcription factor NRF2 can shelter cells and tissues from multiple sources of damage including xenobiotic, electrophilic, metabolic, and oxidative stress. Importantly, the aberrant activation or accumulation of NRF2, a common event in many tumors, confers a selective advantage to cancer cells and is associated to malignant progression, therapy resistance, and poor prognosis. Hence, in the last years, NRF2 has emerged as a promising target in cancer treatment and many efforts have been made to identify therapeutic strategies aimed at disrupting its prooncogenic role. By summarizing the results from past and recent studies, in this review, we provide an overview concerning the NRF2/KEAP1 pathway, its biological impact in solid and hematologic malignancies, and the molecular mechanisms causing NRF2 hyperactivation in cancer cells. Finally, we also describe some of the most promising therapeutic approaches that have been successfully employed to counteract NRF2 activity in tumors, with a particular emphasis on the development of natural compounds and the adoption of drug repurposing strategies.

## 1. Introduction

Living organisms are constantly exposed to multiple challenges and stress sources within the microenvironment and thus have evolved adaptive mechanisms to maintain the homeostasis at the cellular and tissue levels. In this regard, not only fluctuations in the nutrient/oxygen availability but also the presence of electrophiles or xenobiotics can induce alterations in the redox balance and promote cell death by damaging essential macromolecules such as lipids, proteins, and DNA, particularly susceptible to reactive oxygen species (ROS) [1–4]. Traditionally considered as the master regulator of cytoprotective responses against xenobiotic/electrophilic and oxidative stress [5], the transcription factor nuclear factor erythroid 2-related factor 2 (NRF2) was recently found to promote cancer development [6–10], progression [11–14], and therapy resistance [15–22]. Not surprisingly, the renewed interest in NRF2 has fostered many studies directed to elucidate its role in different types of tumors and explore potential therapeutic approaches to prevent or counteract its activation [23–26]. Despite that the dual role of NRF2 as an oncogene or tumor suppressor is still a matter of intense debate [27], in this review, we will mainly focus on its prooncogenic activity while the interested readers are referred to other excellent reviews covering more in detail other aspects [28–31]. We will also briefly discuss risks and benefits derived from the use of negative modulators of NRF2 signaling, with a particular emphasis on repurposing of preexisting drugs and the use of combinatorial treatments aimed at disrupting the redox homeostasis of cancer cells.

## 2. NRF2/KEAP1 Pathway: A Master Regulator of Stress Responses

As already mentioned, the NRF2/KEAP1 pathway is a key cellular defensive mechanism providing protection against environmental challenges caused by electrophiles, oxidants, and xenobiotics. Following its activation, a wide range of stress-related genes is transactivated in order to restore the cellular homeostasis. In the next section, we will describe the structural determinants of NRF2 and its negative regulator KEAP1 that confer redox sensitivity to the system and mediate physical/functional interaction with other regulatory components. We will also briefly discuss the general mechanisms through which the fine-tune regulation of this pathway is exerted and the biological effects prompted by its activation.

### 2.1. NRF2 and KEAP1 Structure

**Figure 1:**
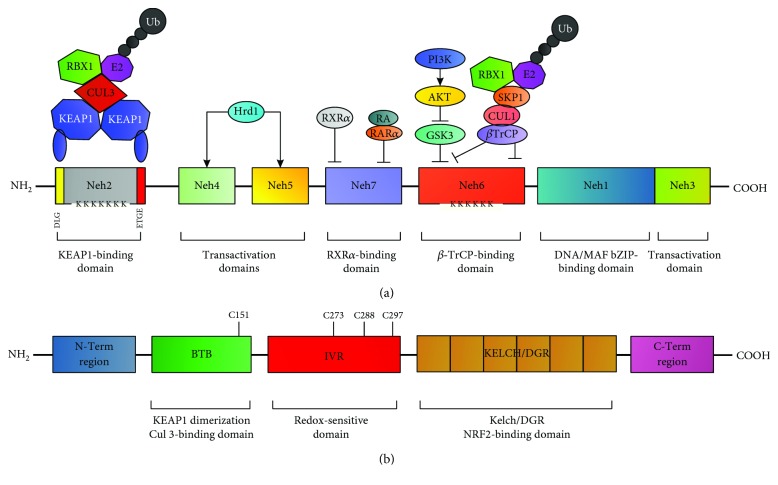
NRF2 and KEAP1 structure/function relationship. (a**)** Schematic representation of the NRF2 structure from *Homo sapiens*. NRF2 is constituted by 7 highly conserved regions, referred to as Neh domains. From the N-term to the C-term, the Neh2 domain contains the DLG/ETGE motifs that are necessary for KEAP1-dependent NRF2 proteasomal degradation and several lysine residues that are directly ubiquitylated by the Cul3/Rbx1/E3 complex; also, a first NLS sequence is localized between the amino acids 42 and 53. The Neh4-5 domains mediate the interaction with Hdr1 and other proteins such as CBP and p300, activating NRF2-dependent transcription; also, NES (between amino acids 191-202) is localized in the Neh5 region. The Neh7 domain contains sites for RXR-*α* and RAR-*α* interaction that induces NRF2 transcriptional repression. The Neh6 domain contains two specific sites of interaction with the ubiquitin ligase *β*TrCP; the binding to the DSGIS motif requires the previous phosphorylation in S344 and S347 by Gsk-3*β* while in contrast, the interaction with the DSPAGS motif is direct. The Neh1 domain possesses the CNC bZIP region, required for DNA binding and dimerization with small MAF proteins and other transcription factors; also, a second NES sequence is localized between amino acids 553 and 562. Neh3 is another transactivation domain containing a second NLS sequence between amino acids 595 and 601. (b**)** Schematic representation of the KEAP1structure from *Homo sapiens*. KEAP1 is composed of 5 domains. The NTR (amino-term region) is followed by the BTB (broad complex, tram-track, and bric-à-brac domain), which is important for KEAP1 homodimerization and interaction with Cul3 and contains a redox-sensitive cysteine residue (Cys151). The next coming domain, known as IVR (intervening region), is a cysteine-rich motif that is particularly sensitive to redox changes and influences KEAP1 function. The next domain, known as DGR (double-glycine repeat), contains six Kelch motifs that promote protein-protein interactions with KEAP1 regulators including NRF2 and other functional partners. Lastly, the CTR (carboxy terminal region) is important for KEAP1-NRF2 interaction.

Human NRF2 is a basic leucine zipper (bZIP) transcription factor belonging to the Cap“n”Collar (CNC) family that was identified as a protein capable of inducing transcription through the binding of the nuclear factor erythroid 2/activator protein 1 (NF-E2/AP-1) motif of the hypersensitive site-2 in the *β*-globin locus control region [32, 33]. Biochemical and structural studies have identified seven highly conserved domains, from Neh1 to Neh7, that are important for NRF2 functions. Among them, Neh1 contains a bZIP domain for DNA and small MAF (*v-Maf* avian musculoaponeurotic fibrosarcoma oncogene homolog) protein binding, Neh2 mediates the interaction with the negative regulator KEAP1 (KELCH-like ECH-associated protein 1) within specific binding sites known as DLG and ETG motifs, and Neh3-5 are required for target genes transactivation and functional interaction with several modulators, while the Neh6 domain contains a serine-rich region that is involved in NRF2 degradation [34] (see Figure 1(a)). The other component of the system, KEAP1, comprises five distinct domains: an N-terminal domain (NTD), a broad complex, tram-track, and bric-á-brac (BTB) homodimerization domain promoting the interaction with the Neh2 domain of NRF2, a cysteine-rich intervening region (IVR), a double-glycine repeat (DGR) containing six Kelch motifs, and a C-terminal region (CTR) [34, 35], both of them required for the association between KEAP1 and NRF2 [36] (see Figure 1(b)).

### 2.2. Mechanisms of NRF2/KEAP1 Pathway Activation and Redox Sensing

**Figure 2:**
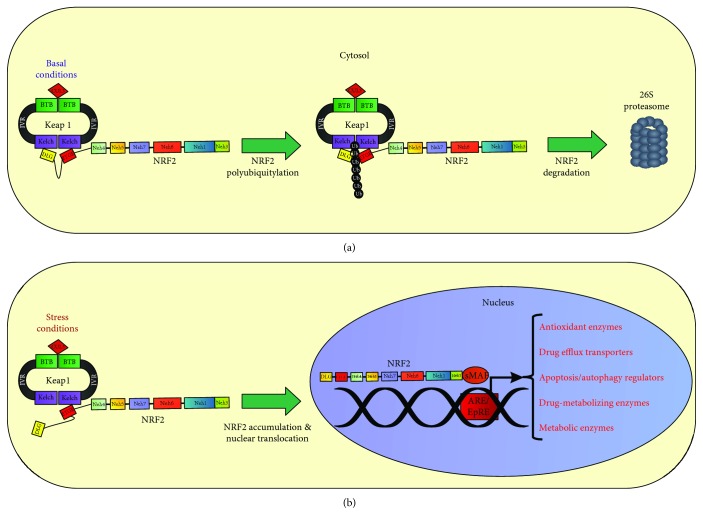
General mechanism of NRF2/KEAP1 control and function. (a**)** Under homeostatic conditions, KEAP1 interacts with NRF2 in the cytosol, promoting its polyubiquitylation and subsequent proteasomal degradation, resulting in minimal or absent NRF2 transactivation. (b**)** In contrast, under different stress conditions, the binding of KEAP1 to NRF2 is strongly impaired, decreasing the likelihood of NRF2 ubiquitylation. As a consequence, a large fraction of NRF2 molecules in the cytosolic pool can translocate into the nucleus, wherein it interacts with small MAF proteins and induces the transcription of several cytoprotective genes.

The fine-tune regulation of the NRF2/KEAP1 pathway depends on the coordinated interaction and activity of multiple components. The current model postulates that under homeostatic conditions, NRF2 interacts with and is complexed by its cytosolic repressor KEAP1, a substrate/binding partner of the Cullin-3- (CUL3-) ring-box 1- (RBX1-) E3 ubiquitin ligase complex that primes NRF2 for proteasomal degradation [37–39] (see Figure 2(a)). Of note, a second pathway controlling NRF2 stability in a KEAP1-independent way has been recently described. Indeed, the protein *β*-TrCP (*β*-transducin repeat-containing protein), a substrate of the Cullin-1- (CUL1-) RBX1-E3 ubiquitin ligase complex with nuclear localization, can recognize and bind to NRF2 upon glycogen synthase kinase-3*β*- (GSK3*β*-) dependent phosphorylation on specific serine residues located in the Neh6 domain, triggering NRF2 ubiquitination and proteasomal degradation [40–42]. Lastly, a third mechanism controlling NRF2 stability has been reported in cirrhotic livers where the protein HRD1, an E3 ubiquitin ligase associated to the endoplasmic reticulum (ER) membranes, is induced by ER stress and interacts with the Neh4-5 domains of NRF2 promoting its degradation [43]. In contrast, the exposure to electrophilic, nitrosative, or oxidative stress promotes NRF2 stabilization (see Figure 2(b)) by various mechanisms [44, 45]. Interestingly, biochemical and structural studies have shown that KEAP1 contains more than 27 cysteine residues with different reactivity and functional impact on NRF2 [46]. Among them, Cys151, located within the BTB domain, was found to facilitate NRF2 activation, while Cys273, 288, and 297, located in the IVR, were found to suppress NRF2 activity facilitating its interaction with KEAP1 [47, 48] (see Figure 1(b)). Similarly, seven highly conserved and redox-sensitive cysteines (Cys119, 235, 311, 316, 414, and 516) have been identified in NRF2 and their oxidative modification was found to prevent KEAP1 recognition and binding [49]. Intriguingly, the existence of a “cysteine code” accounting for divergent responses of NRF2 to specific oxidants seems to be supported by several evidences, although its precise function still needs to be elucidated. Nevertheless, the current model postulates that highly reactive cysteines undergo redox modifications in response to electrophiles and oxidants, inducing a conformational change in KEAP1 that ultimately prevents NRF2 ubiquitination [50–52]. Thus, neosynthesized NRF2 escapes from KEAP1-dependent repression and translocates into the nucleus where it forms heterodimers with sMAF proteins and binds to antioxidant-responsive elements (ARE) or electrophilic-responsive elements (EpRE) within the promoter region of cytoprotective genes, inducing their transactivation [53–56] (see Figure 2(b)). After exerting its function, NRF2 is phosphorylated by the tyrosine kinase FYN that upon GSK-3*β*-dependent activation enters into the nucleus promoting NRF2 retrotranslocation and subsequent cytosolic degradation [57, 58].

### 2.3. Biological Functions Mediated by the NRF2/KEAP1 Pathway

So far, more than 200 target genes of NRF2 have been described in humans [59]. The vast majority of them encode for metabolic enzymes that readily detoxify electrophiles (i.e., phase I/II/III drug metabolism) or scavenge ROS molecules (i.e., antioxidant systems) [60, 61], in order to restore the intracellular redox homeostasis and minimize the oxidative damage [60, 62]. However, increasing evidence indicates that NRF2 can also regulate other biological processes with physiopathological relevance in human diseases (e.g., tumors) such as proliferation [62–67], differentiation [68–72], inflammation [73–76], autophagy [77–81], apoptosis [66, 82–85], mitochondrial function or biogenesis [86–92], and several metabolic pathways involved in iron/heme [32, 93–97], glucose [98–101], glutamine [101–103], lipid [104–107], NADPH [108–110], and pentose phosphate metabolism [111–114]. In the following sections, we will discuss the oncogenic alterations in the NRF2/KEAP1 pathway that confer a selective advantage to malignant cells and their relevance as therapeutic targets in the treatment of cancer.

## 3. NRF2/KEAP1 Prooncogenic Activity in Cancer: Causes and Consequences

The molecular events that lead to cancer initiation, promotion, and progression are characterized by genetic and epigenetic changes in oncogenes and tumor suppressors that control key biological events related to cell proliferation, survival, and metabolism [115–118]. It is now well recognized that cancer cells face many different challenges during their uncontrolled outgrowth such as nutrient withdrawal, hypoxia, and deregulated redox balance, causing the activation of protective mechanisms that ultimately promote adaptation to the microenvironment [119]. Being located at the crossroad of multiple defensive responses influencing cell fate during xenobiotic, oxidative, and metabolic stress, the NRF2/KEAP1 pathway has been the focus of extensive research aimed at elucidating its impact in cancer. In this regard, despite that initial studies recognized its chemopreventive function in carcinogenesis and its cytoprotective role in many human pathologies [120–123], growing evidence also indicates that aberrant activation of the NRF2/KEAP1 pathway is frequently found in many tumors, promoting cancer growth [6, 10, 14], survival [124, 125], metastasis formation [11, 126, 127], and therapy resistance [20, 21, 128–132]. In the following sections, we will describe the molecular mechanisms leading to the activation of prooncogenic NRF2 signaling.

### 3.1. Mutations in the *KEAP1* Gene Induce Hyperactivation of the NRF2/KEAP1 Pathway

**Figure 3:**
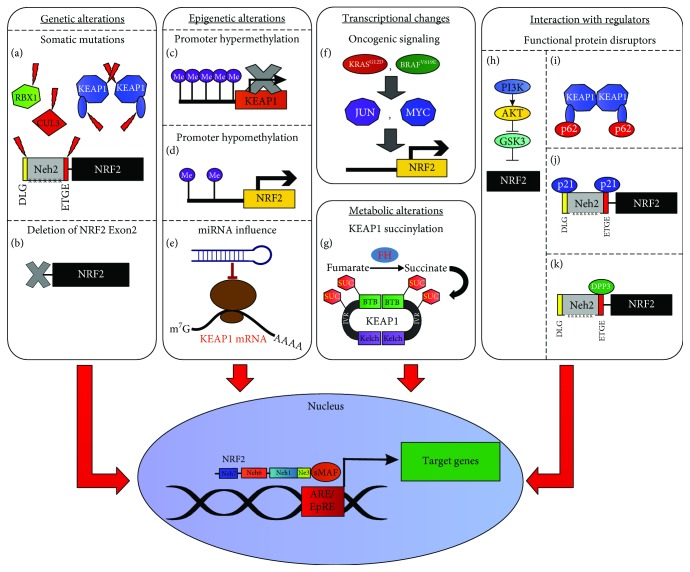
Mechanisms involved in the prooncogenic activation of the NRF2/KEAP1 pathway. Among the genetic alterations so far described, (a) somatic mutations located within specific domains of NRF2 and/or KEAP1 proteins can affect their reciprocal interaction or impair the negative control exerted by other regulators of NRF2 stability such as Rbx1 and Cul3. Also, (b) genetic deletion of NRF2 exon2 can be responsible for impaired NRF2-KEAP1 interaction. In all the cases, NRF2-dependent transcription is potentiated. As concerning the epigenetic changes, (a) either hypermethylation of the CpG islands in the KEAP1 promoter region, (b) the decreased methylation of the CpG islands within the NRF2 promoter, or (c) the inhibition of KEAP1 mRNA translation exerted by certain miRNAs can lead to NRF2 hyperactivation. Moreover, (d) enhanced NRF2 expression can also occur in response to upstream oncogenic signaling triggered by aberrant KRAS and BRAF activation. Also, (g) metabolic changes leading to succinate accumulation can promote KEAP1 succinylation and prevent its interaction with NRF2. Lastly, increased NRF2 nuclear translocation and transactivation can be induced by (h) PI3K-Akt-mediated inhibition of GSK3 or direct interaction with protein regulators such as (i) p21, (j) p62, and (k) dpp3.

The occurrence of genetic mutations in the *NRF2*, *KEAP1*, or *CUL3* genes represents the most frequent and well-characterized mechanism of sustained NRF2 activation in cancer [27] (see Figure 3(a)). In this regard, loss-of-function (LOF) mutations in the *KEAP1* gene, targeting the Kelch/DGR domain, normally required for NRF2 interaction and degradation, were initially identified in tissues or cell lines derived from lung cancer patients [133]. These observations were confirmed in subsequent studies reporting that the biallelic inactivation of *KEAP1* caused by somatic mutations in the Kelch domain or in the IVR region was a frequent event in non-small-cell lung carcinoma (NSCLC). Indeed, LOF mutations in *KEAP1* gene were, respectively, found in 50% (6/12) or 19% (10/54) of the cancer cell lines or cancer samples analyzed, while loss of heterozygosity at 19p13.2, the genetic locus of *KEAP1*, occurred at frequencies of 61% and 41% in NSCLC-derived cell lines and tumor tissues, respectively [134]. On the other hand, genetic sequencing from 65 Japanese patients with lung cancer revealed the presence of five nonsynonymous somatic mutations in 8% of the cases [135]. Notably, in both these studies, the lack of KEAP1 repression was accompanied by constitutive NRF2 activation and increased resistance to chemotherapy. Additional research further expanded the list of *KEAP1* mutations in several cohorts of patients with different subtypes of lung cancer, pointing out the existence of widely distributed alterations beyond the DGR and the IVR motifs of the KEAP1 protein [136–138]. Consistently, all these alterations produced typical clinicopathological features associated with increased NRF2 activity, therapy resistance, and poor prognosis, suggesting that the genetic status of *KEAP1* might be used to stratify NSCLC patients and select personalized therapeutic options [139–142]. In another study, Rekhtman et al. focused on pulmonary large-cell neuroendocrine carcinoma (LCNEC), a heterogeneous group of tumors related to but also distinct from SCLC (small-cell lung carcinoma) and NSCLC. Here, by performing targeted next-generation sequencing (NGS) on 241 cancer genes followed by histopathologic and clinical analyses, *KEAP1*-inactivating mutations were found in 31% of the cases in conjunction with other NSCLC-type alterations of *KRAS*, *STK11*, and *NFE2L2* genes, a molecular signature commonly found in the adenocarcinoma subtype. These findings shed light on the biological origin of LCNEC and might have important implications for the clinical management of these aggressive tumors [143]. Notably, *KEAP1* missense or nonsense mutations have also been reported in malignant melanoma [144] and hepatocellular [145], papillary thyroid [146], and endometrial carcinomas [147] as well as gall bladder [148], breast [149, 150], cervical [151], and ovarian cancers [152]. In all the cases, the inactivation of *KEAP1* was paralleled by NRF2 overexpression that in turn promoted an aggressive phenotype characterized by enhanced antioxidant capacity and decreased sensitivity to chemotherapeutics.

### 3.2. Genetic Alterations in the *NRF2* Gene Lead to Sustained NRF2/KEAP1 Pathway Activation

As concerning the *NRF2* gene, mutations in the DLG/ETGE motifs of the Neh2 domain resulting in decreased KEAP1 binding were also initially identified in biopsies and cell lines from lung cancer [153]. A similar pattern of *NRF2* mutations was also observed in head and neck carcinoma [154], hepatocellular carcinoma [155], and papillary renal cell carcinoma (PRCC) [156] as well as esophageal and skin cancers, resulting in increased malignant potential and chemoresistance [157]. In a recent study, Kerins and Ooi provided a comprehensive dataset of *NRF2* gain-of-function mutations in The Cancer Genome Atlas (TCGA), identifying 226 *NRF2*-mutant tumors from 10364 cases. Overall, somatic mutations were found in 21 out of the 33 tumor types analyzed. Consistently, the vast majority of them occurred in the DLG/ETGE motifs, causing decreased KEAP1 binding and persistent NRF2 activation [158]. Intriguingly, Goldstein et al. reported the first example of increased NRF2 signaling being not caused by somatic mutations, since the genetic deletion of *NRF2* exon 2 (see Figure 3(b)) was found to promote elevated NRF2 activity and stability in head-neck squamous carcinoma (HNSC) and NSCLC, by removing the KEAP1-interacting domain in the absence of other genetic changes [159]. Last but not least, inactivating mutations or copy number loss of the *CUL3* or *RBX1* genes that control NRF2 ubiquitylation/degradation has also been described in PRCC [156] and papillary thyroid [160], esophageal [161, 162], and serous ovarian cancers [163].

In summary, genetic alterations in the NRF2/KEAP1 pathway are one of the leading causes of its prooncogenic activation. Importantly, recent data suggest that somatic mutations are not the solely responsible of aberrant NRF2 signaling since additional genetic changes including copy number variations (CNV) or even the presence of single-nucleotide polymorphisms (SNPs) are expected to emerge as key regulatory events of NRF2 functions in the near future. Intriguingly, despite that the hyperactivation of the NRF2/KEAP1 pathway has been reported in different types of tumors, it appears that this event occurs with higher frequency in certain tissues such as the lungs and the upper aerodigestive tract, presumably due to an increased susceptibility of these sites to various oxidants and chemicals encountered during the lifetime. In this context, NRF2 would confer an augmented detoxification ability that might initially protect the normal cells but also promote malignant progression of already initiated cells, confirming the dual role of the NRF2/KEAP1 pathway. On the other hand, the mutation status of *KEAP1* and *NRF2* genes in NSCLC patients might have a clinical relevance and represent not only a valid predictive biomarker but also a molecular indication for the choice of a personalized therapy [164].

### 3.3. Epigenetic Modulation of *NRF2* and *KEAP1* Genes

Modifications of the epigenetic status in the *NRF2* or *KEAP1* genes have been shown to induce NRF2 stabilization and increased target gene expression in many tumors [165]. For example, the hypermethylation of CpG islands within the *KEAP1* promoter region (see Figure 3(c)) has been reported to induce chemoresistance in malignant glioma [166] and breast [167], prostate [168], colorectal [169], thyroid [160] renal [170], and lung cancers [171–173], due to a marked decrease in the *KEAP1* mRNA levels and an augmented expression of NRF2 target genes. In a recent study, MBD1 (methyl-CpG-binding domain protein 1), a protein highly expressed in pancreatic cancer, was found to downmodulate *KEAP1* expression by influencing the methylation status of its promoter [174]. Also, UHFR1, a well-established epigenetic regulator of DNA methylation status, was found to be highly expressed in human pancreatic ductal adenocarcinoma (PDAC) tissues and associated with large-size tumors. In established PDAC cell lines, UHFR1 was seen to maintain the *KEAP1* promoter in a hypermethylated status, leading to suppression of KEAP1 protein levels and subsequent hyperactivation of the NRF2 antioxidant transcriptional program [175]. Although investigated to a lesser extent, epigenetic changes directly affecting the *NRF2* gene have also been reported (see Figure 3(d)). While decreased NRF2 activity due to hypermethylation of the *NRF2* promoter has been observed in prostate cancers [176, 177], its hypomethylation was recently reported in colorectal cancer, an event associated to *NRF2* overexpression and augmented chemoresistance [178, 179]. In a study from Li and colleagues, the reduced expression of the methyl transferase EZH2 caused a decrease in the trimethylation of lysine 27 on histone H3 (H3K27Me3) in the *NRF2* promoter region, repressing *NRF2* expression and NSCLC progression both *in vitro* and *in vivo* [180]. Also, the epigenetic sensor BRD4 (bromodomain protein 4), an acetylated histone-binding protein implicated in transcriptional regulation, was found to regulate KEAP1 function in a model of prostate cancer. Indeed, under unstressed conditions, BRD4 was able to activate the *HMOX1* promoter through the SP1 promoter-binding sites in a NRF2-independent manner, a mechanism causing also sustained *HMOX1* transcription during oxidative stress, despite that *KEAP1* expression was surprisingly enhanced by BRD4. The authors postulated that the two-sided regulatory mechanism of BRD4 might prevent prostate cancer cells from a loss of *HMOX1* promoting cell survival during oxidative stress. Thus, BRD4 might represent a fine-tune modulator of the antioxidant response in prostate cancer by influencing the expression of HO-1 and interacting with the NRF2/KEAP1 network [181].

### 3.4. Regulation of NRF2 Activation by miRNAs

Importantly, an emerging mechanism of *NRF2/KEAP1* epigenetic deregulation in cancer is represented by microRNAs (miRNAs), small noncoding molecules that recognize the 3′-untranslated regions (UTRs) of specific mRNAs and negatively regulate their abundance by translation blocking or forced degradation [182–184] (see Figure 3(e)). With this respect, several miRNAs with potential regulatory effects on the NRF2/KEAP1 pathway were initially identified using bioinformatic tools [185]. Among them, miR-28 was recognized as the first miRNA to negatively modulate *NRF2* in the MCF-7 breast cancer line [186]. Similarly, miR-507, miR-634, miR-450a, and miR-129-5p were found to directly inhibit *NRF2* expression while their low levels were associated with poor outcome in esophageal carcinoma [187]. In another report, miR-340 was shown to mediate Cisplatin resistance in HepG2 cells, since this drug induced opposite changes in miR-340 levels and *NRF2* expression that could be substantially reverted by miR-340 mimics [188]. Several studies have also focused on miR-144. Indeed, miR-144-3p was found to be increased in the peripheral blood and in the bone marrow of AML (acute myeloid leukemia) patients compared to controls and also in leukemia HL-60 cells, being its inhibition sufficient to promote apoptosis and suppress NRF2 activation [189]. In hepatocellular cancer (HCC) cell lines, NRF2 levels were found to be negatively regulated by miR-144, whose ectopic expression enhanced 5-FU cytotoxicity [190] while in neuroblastoma SH-SY5Y cells, the use of 144 mimics induced ROS-dependent apoptosis by decreasing the expression of enzymes involved in GSH synthesis and GSH-dependent ROS scavenging [191]. In the same cell line, miR-153, miR27a, and miR-142-5p were also found to repress NRF2-dependent transactivation of cytoprotective genes while the forced expression of each miRNA was sufficient to markedly decrease the levels of GCLC and GSR [192]. Recently, miR-155 inhibition was found to attenuate the malignancy and promote apoptosis in arsenic-transformed bronchial epithelial cells by repressing *NRF2*, suggesting that miR-155 might promote malignant transformation of lung cells exposed to arsenite [193]. Similarly, miR-153 and miR-93a were also proposed to drive breast carcinogenesis, since their increased expression was paralleled by reduced NRF2 protein content in mammary tumors and breast cancer cell lines treated with 17*β*-estradiol [194, 195]. Hence, several miRNAs that directly target and suppress NRF2 function have been so far described, although additional studies are required to assess the potential impact of their modulation in cancer therapy. Last but not least, new exciting studies suggest that NRF2 can in turn regulate the expression of several miRNAs in tumors, pointing out the existence of complex cross-regulatory interactions between these two systems in human malignancies [85, 114, 196].

### 3.5. Modulation of KEAP1 Function by miRNAs

As concerning KEAP1, miR-141 was the first identified miRNA to directly repress its levels in ovarian carcinoma cell lines [187] while the same miRNA was later shown to confer 5-FU resistance in HepG2 cells, an alteration phenocopied by miR-141 mimics and partially reversed by the reintroduction of KEAP1 [197]. Recently, miR-432-3p was found to positively modulate NRF2 activity by impairing *KEAP1* mRNA translation in esophageal squamous cell carcinoma (ESCC), being its overexpression associated to increased Cisplatin resistance, while conversely, its genetic depletion by CRISPR/Cas9 restored the chemosensitivity [198]. By using miRNA arrays, Eades and coworkers found that forced reexpression of miR-200a, normally repressed in breast cancer cells [199], was able to impair *KEAP1* mRNA translation, inducing NRF2-mediated NQO1 transactivation [200]. In another context, increased expression of miR-200a was shown to negatively modulate KEAP1 levels in response to methylseleninic acid (MSA), while the use of antagomir-200a attenuated the KEAP1 downregulation induced by MSA in ESCC cells [201]. Moreover, a direct targeting of the 3′-UTR in the *KEAP1* mRNA by the miR-7 was recently demonstrated in neuroblastoma SH-SY5Y cells, where its high expression was seen to increase the NRF2-dependent transcription of the antioxidant genes *HMOX1* and *GCLM*, therefore attenuating methyl-4-phenylpyridinium- (MPP+-) induced toxicity [202]. Lastly, in a very recent study from Qu and coworkers, miR-148b was found to be highly expressed in normal endometrial tissues but weakly represented in endometrial cancerous tissues. Here, in RL95-2 human endometrial cancer cells, the overexpression of miR-148b was shown not only to markedly decrease cell proliferation but also to enhance ROS production, due to the repression of *HIF1* and *NRF2* expression resulting from EMRP1 (endoplasmic reticulum metalloprotease 1) downregulation [203].

In summary, the existence of multiple epigenetic mechanisms controlling the NRF2/KEAP1 pathway opens new exciting opportunities for therapeutic manipulations of NRF2 oncogenic signaling not related to its direct pharmacologic inhibition, although additional research is needed to better clarify potential risks and benefits of this approach in cancer patients.

### 3.6. Protein Interactors that Modulate the NRF2/KEAP1 Pathway: The Role of p21

Compelling evidence indicates that several proteins can promote NRF2 hyperactivation by directly disrupting its interaction with KEAP1 [204] (see Figures 3(h), 3(j), and 3(k)). Initial studies focused on p21 (CIP1/WAF1), a cyclin-dependent kinase inhibitor (CDKi) that under mild oxidative stress conditions is induced by p53, promoting cell cycle arrest and DNA repair until the intracellular redox homeostasis is restored [205–207]. Chen and coworkers reported that p21 was able to recognize and directly bind the DLG/ETGE motifs of NRF2, preventing KEAP1 interaction and subsequent NRF2 ubiquitination (see Figure 3(j)), therefore promoting increased *NQO1* and *HMOX1* expression [208]. Recent data indicate that NRF2 can in turn promote *p21* expression in A549 cells, through direct binding on highly conserved sites within the *p21* promoter [209] or by indirect modulation prompted by SP1 recruitment and platelet-derived growth factor A- (PDGFA-) dependent activation of the AKT/p21 pathway in HCC [14].

### 3.7. Modulation of the NRF2/KEAP1 Pathway by the Autophagy Regulator p62

On the other hand, extensive research has been pursued on p62, also known as sequestosome 1 (SQSTM1), a protein primarily involved in the activation of autophagy that directs polyubiquitinated proteins and damaged organelles to lysosomal degradation [210]. Many studies have shown that once phosphorylated by upstream events, p62 can directly bind to KEAP1 through the so-called KEAP1 interaction region (KIR) (346-KEVDPSTGELQSLQ-359), causing NRF2 displacement and its stabilization [211–214] (see Figure 3(i)). Of note, persistent phosphorylation of p62 has been reported in hepatic adenoma of liver-specific autophagy-deficient mice [215] as well as in human HCC positive for hepatitis C, where it was found to promote metabolic reprogramming by increased NRF2 activation [216]. A role for p62 in HCC carcinogenesis and progression was also confirmed in three independent studies. In one case, the increased levels of phospho-p62 were found to be associated with NRF2 activation in biospecimens derived from 30 HCC patients [217]; in another case, p62 was found to be upregulated in preneoplastic lesions and both necessary/sufficient for HCC induction in mouse models [218], while in the last study, p62 expression elicited by ferroptosis inducers was able to inactivate KEAP1, promoting NRF2 stabilization and transactivation of both *NQO1* and *HMOX*1 genes [219]. Similarly, a regulatory role of p62 in the NRF2/KEAP1 pathway was also observed in the context of breast carcinoma. Indeed, an earlier study reported that high levels of p62 were significantly correlated with *HER2* overexpression in human breast cancers [220], while a role for p62 in breast carcinogenesis was further evidenced in a recent study wherein p62 was shown to facilitate *HER2*-dependent mammary tumorigenesis in MMTV-*Neu* transgenic mice by the activation of multiple pathways, including the NRF2/KEAP1 [221]. Also, altered *p62* expression was associated to increased NRF2 activation and enhanced chemoresistance in cancer stem cell- (CSC-) enriched mammospheres derived from MCF-7 breast cancer cells, compared to monolayer cultured cells [222]. More recently, the same group showed that high levels of CD44, the most common marker of CSC, led to p62-dependent NRF2 activation in breast CSC-like cells, promoting an aggressive phenotype with sustained tumor growth and increased drug resistance [223]. In another context, it has been proposed that p62 might serve as a prognostic marker in patients with glioma, being its content positively correlated to the NRF2 levels and a poor prognosis [224]. From a clinical perspective, p62 was also found to decrease arsenic sensitivity in human transformed lung bronchial epithelial BEAS-2B cells, by noncanonical stimulation of the NRF2/KEAP1 pathway [225], presumably due to its constitutive activation after carcinogenesis induction [226]. Similar observations were also made in human ovarian cancer cells (HOCCs), wherein p62-dependent activation of NRF2 was proposed to increase the expression of antioxidant genes leading to Cisplatin resistance [227]. Notably, p62 was also found to represent a target of NRF2 which might in turn promote its persistent activation through the induction of a positive feedback loop in the p62/NRF2/KEAP1 pathway [228]. In this regard, the aberrant activation of this axis was also found to decrease lung cancer sensitivity to isodeoxyelephantopin (ESI) due to HO-1 upregulation [77] or mediate proteasome inhibitor resistance in multiple myeloma cells through redox, metabolic, and translational reprogramming [229]. These data suggest that pharmacologic targeting of this protective pathway might represent a valid anticancer strategy, especially in conjunction with prooxidizing drugs [230]. Also, since amplification of the *p62* gene or aberrant accumulation of its phosphorylated form is frequently found in many cancers, either inhibitors of p62 phosphorylation or antagonists of p62/NRF2 interaction might restore the route of NRF2 proteasomal degradation in the context of a functional *KEAP1* expression.

### 3.8. Other Protein Regulators of the NRF2/KEAP1 Pathway

Several other proteins containing an ETGE motif have been identified as positive regulators of NRF2 function by preventing KEAP1 association and subsequent NRF2 ubiquitination. For example, the WTX tumor suppressor protein (Wilms tumor gene on the X chromosome) was found to bind KEAP1 and induce NRF2 stabilization in HEK293 cells [231]. Similarly, PALB2 (partner and localizer of BRCA2), a binding partner of BRCA2 (breast cancer type 2 susceptibility protein), was found to directly interact with KEAP1 and induce NRF2 nuclear accumulation followed by antioxidant gene expression in HEK293 and U2OS cells [232]. Also, Hast and coworkers reported that the protein dipeptidyl peptidase 3 (DPP3) can promote NRF2 stabilization by sequestering KEAP1 in HEK293T and lung adenocarcinoma H2228 cells [233] (see Figure 3(k)). Collectively, these data suggest that the overexpression of different types of ETGE-containing proteins in cancer cells might sustain NRF2 activity even in the absence of activating mutations in the *NRF2* or inactivating mutations in the *KEAP1* genes.

### 3.9. Metabolic Regulation of the NRF2/KEAP1 Pathway

It is becoming increasingly clear that some metabolic intermediates can also hyperactivate NRF2, disrupting its interaction with its negative regulator KEAP1. With this respect, the lack of fumarate hydratase (FH), an enzyme that converts fumarate to malate in the TCA (tricarboxylic acid) cycle, was found to promote fumarate accumulation and lead to succinylation of KEAP1 cysteines, NRF2 stabilization, and subsequent transactivation of stress-related genes (see Figure 3(g)) in PRCC [234, 235].

### 3.10. Functional Interaction with Oncogenic Signaling

#### 3.10.1. Activation of the NRF2/KEAP1 Pathway Induced by K-RAS and B-RAF

Accumulating evidence suggests that other cancer-specific alterations, particularly those related to oncogenic signaling can strongly influence the activity of NRF2 without affecting its protein stability but rather increasing its mRNA levels (see Figure 3). With this respect, initial studies showed that the oncogenic activation of K-RAS and B-RAF was sufficient to increase the *NRF2* mRNA levels and promote ROS detoxification (see Figure 3(f)) in human pancreatic cancer cells but also in primary cells and tissues of mice expressing either of the transgenic alleles [10]. Likewise, the constitutively active form of K-RAS (G12D) was found to promote *NRF2* transcription and chemoresistance through the MEK/ERK pathway in NSCLC cells and in a murine model of lung cancer, being the effects at least partially reverted by coadministration of the NRF2 inhibitor Brusatol [236]. Active HRAS (V12) was also found to induce *HO-1* overexpression and mediate apoptosis resistance in renal cancer cells, abrogated by *NRF2* knockdown or ERK inhibitors [237]. Moreover, aberrant activation of NRF2 target genes (i.e., *ABCC1*) has been shown to occur in human oropharyngeal carcinoma KB-7D cells due to B-RAF-mediated *NRF2* gene transcription and histone acetyl transferase- (HAT-) dependent NRF2 acetylation, promoting Etoposide resistance [238]. More recently, by using an inducible form of activated K-RAS (G12V), Shao and coworkers demonstrated that the downstream oncogenic signaling was able to induce *NRF2* expression in several cancer cells both *in vitro* and *in vivo*. Importantly, increased expression of the antioxidant genes *NQO1* and *HMOX1* was found to promote an aggressive phenotype associated to chemoresistance, while the genetic ablation of *NRF2* by CRISPR/Cas9 was able to impair the malignant progression and restore the sensitivity to several anticancer drugs [239]. Therefore, targeting NRF2 might represent a valid therapeutic strategy in solid tumors with aberrant activation of the K-RAS signaling associated to an aggressive behavior and chemoresistance.

#### 3.10.2. Regulation of the NRF2/KEAP1 Pathway by PI3K/AKT Signaling

Importantly, strong evidence also indicates that aberrant activation of the PI3K/AKT pathway, a master regulator of cancer cell growth, survival, and metabolism [240, 241] can act upstream NRF2 signaling in different types of tumors (see Figure 3(h)). Initial studies provided indirect evidence of a functional interaction between the PI3K/AKT and NRF2/KEAP1 pathways since the pharmacologic inhibition of the former was able to prevent NRF2 nuclear accumulation in renal adenocarcinoma cells [242] and auditory cells [243]. Later studies conducted on lung cancer cell lines with *KEAP1* (A549 and H2126) or *NRF2* (EBC1 and LK2) mutations demonstrated that the sustained activation of the PI3K/AKT pathway was accompanied by increased *NRF2* mRNA levels and NRF2 nuclear accumulation leading to metabolic reprogramming, enhanced cell proliferation, and apoptosis evasion [101]. Noncanonical NRF2 activation by the PI3K/AKT signaling was also observed in HCT-116 colorectal cancer cells treated with benzyl isothiocyanate (BITC), an aromatic compound known to induce the accumulation of NRF2 and other autophagic molecules, since when PI3K/AKT inhibitors were coadministered, the sensitivity of HCT-116 cells to BITC was greatly enhanced [244]. Importantly, two independent studies reported that NRF2 can be a downstream target of PI3K/AKT activation also in breast cancer. More in detail, the estrogen E2 was found to increase the expression of NRF2-dependent antioxidant genes not only in MCF-7 breast cancer cells [245] but also in normal or malignant *BRCA1*-deficient cells through the activation of the PI3K/GSK3*β* pathway [246]. Collectively, these data provide evidence of the complex interrelation between oncogenic signaling and the NRF2/KEAP1 pathway shedding light on multiple mechanisms of noncanonical NRF2 activation. Of note, since anticancer drugs targeting the oncogenic pathways that lie upstream of NRF2 are currently used in cancer treatment, their repurposing might represent a valid strategy to also hamper NRF2 activation. On the other hand, the combination with other compounds that directly inhibit NRF2 transcriptional activity or interfere with its downstream effectors is expected to synergize with already established chemotherapeutics and potentiate their efficacy.

### 3.11. Stress Cues in the Microenvironment

#### 3.11.1. Proinflammatory Stimuli Leading to NRF2/KEAP1 Pathway Activation

As already mentioned, the tumor cells encounter adverse conditions during their malignant progression including oxidative, xenobiotic, and metabolic stress [39]. Despite that these stimuli are well-known and prototypical inducers of the NRF2/KEAP1 pathway, accumulating evidence indicates that proinflammatory conditions or nutrient withdrawal can also activate NRF2 in cancer cells. In this regard, lipopolysaccharide (LPS) and other Toll-like receptor (TLR) agonists were found to trigger NRF2 signaling through p62-dependent KEAP1 degradation in murine RAW264.7 macrophages [247] or in THP-1 human monocytes, wherein increased *NRF2* mRNA levels, NRF2 protein accumulation, and transactivation were caused by NF-*κ*B-mediated signaling [248]. More recently, LPS was reported to induce NRF2 activation in a murine model of acute lung injury as well as in NSCLC A549 cells [249]. Also, NF-*κ*B was found to drive constitutive *NRF2* expression in human AML cells, promoting chemoresistance to several cytotoxic drugs [250].

#### 3.11.2. Energetic Changes that Modulate the Activity of the NRF2/KEAP1 Pathway

Interestingly, changes in the cellular energy status can also modulate the NRF2 signaling. Indeed, the AMP-activated protein kinase (AMPK), a well-known sensor of the energetic stress [251], was shown to promote NRF2 phosphorylation on Ser550, facilitating its nuclear accumulation and subsequent activation [252]. Intriguingly, while earlier studies reported that the AMPK activator AICAR could induce NRF2 and modulate the redox status of HCC cells independently from AMPK function [253], more recent data indicate that in many cancer cells, the AMPK activity is in contrast required for the expression of NRF2-dependent antioxidant genes in response to glucose withdrawal [254]. Similarly, glucose deprivation was also shown to promote NRF2-mediated induction of antioxidant enzymes in both MCF-7 and T47D breast cancer cells, independently from the macroautophagic response elicited by p62 degradation, since the autophagy inhibitor chloroquine could not prevent the expression of *NQO1* [255]. These evidences suggest that NRF2 might constitute a molecular link between energy sensing and redox regulation in several tumors and therefore represent an attractive therapeutic target.

#### 3.11.3. Components of ER Stress Response Can Regulate the NRF2/KEAP1 Pathway

Many studies have also tried to elucidate the potential role of ER stress in the regulation of the NRF2/KEAP1 pathway, since this condition is frequently found in cancer cells exposed to nutrient deprivation, hypoxia, radiotherapy, and chemotherapy. With this respect, by comparing cancer cell lines with different sensitivities to ER stress, Salaroglio and coworkers have shown that the ER-resistant cells also acquire a multidrug-resistant (MDR) phenotype due to higher expression of the UPR (unfolded protein response) sensor protein kinase RNA-like endoplasmic reticulum kinase (PERK) that in turn promotes NRF2-dependent MRP1 (multidrug resistance-associated protein 1) transcription. Importantly, disruption of the PERK/NRF2 axis was able to reverse both the resistance to ER stress and to anticancer drugs [256]. In another study focused on prostate cancer cells, the glucose-regulated protein of 78 kD (GRP78), a key molecular chaperone in the ER, was seen to promote noncanonical NRF2 activation in response to the ER stress inducer tunicamycin, without appreciable ROS production [257]. Activation of the GRP78/PERK/NRF2 axis was also found to mediate ROS-independent but ER stress-dependent *NRF2* induction, an event necessary to maintain low ROS levels and the stemness of cancer-initiating cells [72]. Also, recent data indicate that the fibroblast growth factor 19 (*FGF19*), a gene frequently amplified in HCC, can activate a cytoprotective response against ER stress by triggering a FGFR4/GSK3*β*/NRF2 signaling cascade in cultured HCC cells and in a xenograft mouse model [258]. Intriguingly, a very recent study on multiple myeloma (MM) provided evidence that NRF2 can in turn regulate the ER stress response. Indeed, constitutive NRF2 activation was detected in almost 50% of MM primary samples and in several MM cell lines and associated to resistance towards proteasome inhibitors (PI), while NRF2 repression was conversely accompanied by upregulation of the ER stress response protein CHOP and restored sensitivity to PI treatment [83].

Taken together, these data highlight the existence of a complex interrelation between the NRF2/KEAP1 pathway and stress-related responses commonly found in the microenvironment of malignant tumors, confirming that aberrant NRF2 activation can represent a common feature of cancer cells, even in the absence of alterations of the redox status or prooncogenic mutations in *NRF2*/*KEAP1* genes. Importantly, since many tumors rely on the NRF2-mediated cytoprotective response to counteract adverse conditions, the pharmacologic targeting of NRF2 would presumably potentiate the efficacy of anticancer treatments that promote cell death through the induction of different types of stress.

## 4. Therapeutic Strategies for NRF2 Inhibition in Cancer

It is well recognized that NRF2 hyperactivation can be induced by several mechanisms in cancer cells, with profound implications in tumor biology. Being at the intersection of multiple oncogenic and cytoprotective pathways, NRF2 can play a direct or indirect role in each of the cancer hallmarks so far described, including carcinogenesis, sustained proliferation, apoptosis evasion, metabolic reprogramming, altered redox balance, metastasis formation, and therapy resistance [259]. With this respect, it is known that the consequent elevation of drug-metabolizing enzymes, efflux transporters, and redox-modulating proteins represents a clinically relevant obstacle, largely protecting cancer cells from drug treatment, radiotherapy, and various apoptotic inducers [260]. In particular, a growing body of evidence indicates that several drug efflux transporters controlled by NRF2 represent crucial determinants of therapy resistance in many tumors. For example, aberrant NRF2 activation has been shown to induce overexpression of the *MDR1* (multidrug resistance protein 1), *MRP1-5* (multi-drug resistance-associated protein 1-5), and *BCRP* (breast cancer resistance protein) genes or to increase the activity of their corresponding proteins, leading to widespread chemoresistance [261–266]. Therefore, there is a growing interest in the development of effective therapeutic strategies that might disrupt the oncogenic functions of NRF2. In line of the principle, pharmacologic targeting of the NRF2/KEAP1 pathway with antineoplastic purposes can be achieved by two different strategies, the first being based on the positive modulation of KEAP1 and the second on the inhibition of NRF2 (see Table 1). Additionally, direct or indirect modulation of upstream and downstream functional interactors can be exploited (see Table 1). These approaches will be described in the following sections.

### 4.1. Inhibitors of NRF2

#### 4.1.1. High-Throughput Screening (HTS)

High-throughput screening (HTS), particularly when combined with cell-based assays, is increasingly recognized as a valuable approach not only in the discovery of new potential anticancer drugs but also in the identification of the novel therapeutic use for many compounds already approved by the FDA [267]. Therefore, not surprisingly, several studies have taken advantage of this tool to uncover negative modulators of NRF2 activity. For example, by using high-throughput screening to identify small molecule inhibitors of the NRF2 transcriptional activity at ARE sites, AEM1 was found to broadly impair the expression of NRF2 target genes, leading to growth inhibition and increased chemosensitivity of A549 NSCLC cells *in vitro* and *in vivo* [268] (see Table 1). Also, a quantitative high-throughput screening on ∼400 000 small molecules made by Singh and coworkers led to the identification of ML385, a compound with high specificity and selectivity for NSCLC with constitutive NRF2 activation caused by inactivating mutations of *KEAP1* (see Table 1). In preclinical models of NSCLC, the combined use of ML385 with Carboplatin was associated to significant antitumor activity, confirming that NRF2 targeting is a promising strategy for the treatment of advanced NSCLC [25]. More recently, Matthews and coworkers screened two commercially available libraries of known biologically active small molecules, an RNAi library targeting the majority of the druggable genome and a small collection of natural products from marine cyanobacteria. This led to the identification of cardiac glycosides, STAT3 inhibitors, and actin-disrupting agents, with the ability to attenuate NRF2 activity and synergize with chemotherapeutic agents in NSCLC A549 cells. Moreover, novel putative NRF2 targets including the transcription factors TWIST1 and ELF4, the protein kinase NEK8, the TAK1 kinase regulator TAB1, and the dual-specific phosphatase DUSP4 were also identified, expanding the list of potential molecular targets for effective NRF2 inhibition [269].

#### 4.1.2. Natural Compounds with Inhibitory Effects on NRF2

The therapeutic properties of natural compounds derived from medicinal plants have been known for decades and successfully employed to treat a great variety of human diseases. Recently, many phytochemicals and other plant extracts have emerged as promising anticancer agents and are currently under clinical trial investigation or already administered in the established therapeutic regimens. Extensive research has been recently pursued with the specific intent of finding natural compounds with inhibitory properties on NRF2 functions. In this section, we will describe some of the most recent and significant discoveries in this field.

*(1) The Use of the Procyanidin CCE*. In a research from the group of Hiratsuka, it was found that procyanidins (condensed tannins) prepared from *Cinnamomi cortex* extract (CCE) can suppress NRF2-regulated activity and *NRF2* expression in human A549 NSCLC cells [270] (see Table 1), an observation confirmed in a later study from the same authors, wherein the treatment of cancer cell lines of different origin with CCE was shown to selectively reduce the *NRF2* mRNA levels and suppress cell proliferation only in the presence of *NRF2* overexpression [271]. Intriguingly, a novel mechanism of CCE procyanidin-dependent NRF2 repression was reported more recently by the same authors since the treatment of A549 cells with this compound led to IGF1R (insulin-like growth factor 1 receptor) phosphorylation and proteasome-independent but cysteine protease-dependent NRF2 degradation [272] (see Table 1). Therefore, the use of CCE procyanidin might represent a valid strategy to impair NRF2 signaling in those tumors wherein this transcription factor is expressed at high levels or constitutively active.

*(2) The Flavonoid Luteolin*. It has been also reported that Luteolin, a flavonoid present in food plants and vegetables, can potently inhibit NRF2 in A549 NSCLC cells (see Table 1), increasing their sensitivity to several anticancer drugs [273], an observation that the same group further confirmed *in vivo* by xenografting A549 cells in athymic nude mice. In that context, the oral administration of Luteolin was able to strongly impair the growth of xenograft tumors, decreasing cell proliferation, *NRF2* expression, and antioxidant gene transactivation. Furthermore, Luteolin enhanced the anticancer effect of Cisplatin, demonstrating that this natural compound can potentially act as an adjuvant in the chemotherapy of NSCLC [274] (see Table 1).

*(3) The Alkaloid Trigonelline*. Some studies have also focused on Trigonelline, an alkaloid that is abundantly present in many plants like coffee beans, garden peas, hemp seed, oats, and fenugreek seed. In this regard, Arlt and coworkers showed that in PDAC cell line (MiaPaca2, Panc1, and Colo357) high basal NRF2 activity conferred protection from Etoposide- or TRAIL-induced apoptosis by increasing the expression of proteasomal genes. Notably, submicromolar doses of Trigonelline efficiently suppressed NRF2 nuclear accumulation and the proteasome activity, abrogating their protective effects *in vitro* and *in vivo* [275] (see Table 1). Therefore, the use of Trigonelline might be beneficial in patients affected by pancreatic cancer for which limited options are currently available.

*(4) The Quassinoid Brusatol*. Among the other compounds, extensive research has focused on Brusatol, a quassinoid plant extract from *Brucea javanica*, traditionally used in Chinese medicine for treating various diseases including cancer, amoebic dysentery, and malaria [276–278]. So far, many investigations have been conducted to better explore the biological effects of Brusatol in cancer. Earlier studies reported that extracts from the *Fructus Bruceae* plant, including brucein D, exhibited potent antitumor activity on pancreatic adenocarcinoma cell lines despite the lack of mechanistic explanations [279, 280]. Later on, Ren and coworkers demonstrated that Brusatol was able to strongly potentiate the cytotoxic effect of Cisplatin in a broad range of cancer cell lines and A549 NSCLC xenograft, by enhancing the ubiquitination of NRF2 and its subsequent degradation [281] (see Table 1). The potential use of Brusatol in NSCLC treatment was subsequently confirmed *in vivo* by Tao et al., since in a mouse model of K-RAS-G12D-induced lung cancer, Brusatol was shown to enhance the antitumor effects of Cisplatin, leading to a decreased tumor burden and improved survival [236]. Later studies conducted on mouse Hepa-1c1c7 hepatoma cells and primary human hepatocytes consistently reported that Brusatol could transiently and rapidly deplete the NRF2 protein levels in a KEAP1-independent way through a posttranscriptional mechanism, strongly increasing the cell sensitivity to electrophilic stress inducers [282] (see Table 1). Also, Brusatol has been proposed to act as a natural sensitizer of NSCLC to radiotherapy, since even nanomolar doses of this compound were sufficient to enhance the responsiveness of A549 NSCLC cells to irradiation, inducing extensive DNA damage [283] (see Table 1). In another study conducted on mammospheres derived from MCF-7 and MDA-MB-231 breast cancer cells, Brusatol decreased the NRF2 protein levels and enhanced the cytotoxicity of Taxol, leading to intracellular ROS accumulation [284] (see Table 1). Other research provided evidence that Brusatol inhibits growth and induces apoptosis in PATU-8988 and PANC-1 pancreatic cancer cells, through the activation of the JNK (c-Jun N-terminal kinase)/p38 MAPK (mitogen-activated protein kinase) and subsequent inhibition of NF-*κ*B/STAT3/BCL2 signaling [285] (see Table 1). These observations were further confirmed in a very recent study from the same group wherein Brusatol was found to reduce the NRF2 protein content in a KEAP1-independent way and to decrease the expression of genes related to the MDR family involved in Gemcitabine resistance of pancreatic cancer cells. Importantly, Brusatol also promoted an increase of the intracellular ROS levels, indicating that both the mechanisms can contribute to revert the chemoresistant phenotype of pancreatic cancers [286] (see Table 1). Strikingly, Brusatol was also found to exert biological effects beyond the downmodulation of the NRF2/KEAP1 pathway in other types of tumors. Indeed, by using HCT116 colon carcinoma cells, Lu and coworkers showed that Brusatol can suppress the HIF1*α* accumulation under hypoxia and abrogate the HIF-dependent transactivation of target genes involved in glucose metabolism and angiogenesis, by promoting HIF1*α* degradation and decreased ROS production in the cytosol and mitochondria [287]. Of note, two independent studies focused on Brusatol's mode of action revealed that its effects are not derived from direct and specific inhibition of NRF2 but rather are caused by the suppression of both cap-dependent and cap-independent protein translations, arguing against a possible use in cancer therapy due to potential off-target effects [288, 289]. However, despite this seemingly disqualifying observation, additional research has been pursued and the antitumor effects of Brusatol were also confirmed in a model of colorectal cancer (CRC). In this regard, by using tissue microarrays, Evans and coworkers found that NRF2 was highly expressed in primary CRC and metastatic tissues compared to normal colon. Here, siRNAs against *NRF2* or Brusatol were found to induce cell death in human (HCT116) and murine (CT26) cell lines, enhancing also the toxicity of Irinotecan, while Brusatol potently abrogated CRC tumor growth in subcutaneously and orthotopically allografted syngeneic mice [290] (see Table 1). In another very recent study, the cotreatment with Brusatol and UVA led to the inhibition of A375 melanoma cell proliferation triggering ROS-dependent apoptosis. Furthermore, decreased *NRF2* expression was shown to attenuate colony formation and tumor development from A375 cell xenograft in heterotopic murine models, supporting the notion that the combined use of Brusatol and UVA might offer a valuable therapeutic option against malignant melanoma through the disruption of the tumor antioxidant defenses [291] (see Table 1). Importantly, apart from the antitumor effects on solid tumors, the chemosensitizing properties of Brusatol were also recently confirmed in hematological malignancies by Karathedath and colleagues. Here, Brusatol was found to potentiate the cytotoxic effects induced by individual administration of Cytarabine (Ara-C), Daunorubicin (Dnr), and arsenic trioxide (ATO) in several AML cell lines [17] (see Table 1). Therefore, these studies collectively support the notion that NRF2 could be an ideal target in solid and also in hematologic tumors, while Brusatol might provide clinical benefit especially when combined with anticancer drugs that stimulate ROS production, a strategy also applicable to therapy-resistant forms.

*(5) The Flavonoid Chrysin*. Other studies focused on HCC explored the potential antitumor activity of Chrysin, an active natural bioflavonoid known to protect against carcinogenesis. Here, Chrysin was found to decrease the mRNA and protein levels of NRF2 and to chemosensitize multi-drug-resistant HCC-derived cells (Bel-7402/ADM) to Doxorubicin, by preventing *HO-1* expression due to the downmodulation of the PI3K/AKT/ERK pathways [292] (see Table 1). Later research extended these observations to human glioblastoma, since Chrysin was found to inhibit the proliferation, migration, and invasiveness of glioblastoma cells by decreasing NRF2 nuclear translocation and suppressing the expression of both *HO-1* and *NQO1*. Moreover, while NRF2 shRNA attenuated the observed antitumor effects in several glioblastoma cell lines, Chrysin decreased the phospho-ERK1/2 protein content and inhibited tumor growth in U87 xenografts [293] (see Table 1). These results suggest that Chrysin might have a potential application as a natural sensitizer in the chemotherapy of glioblastoma and HCC.

*(6) The Flavonoid Apigenin*. Other research focused on Apigenin, a common dietary flavonoid that is highly abundant in many fruits, vegetables, and Chinese medicinal herbs. Although its anti-inflammatory, antioxidant, antibacterial, and antiviral properties have been long-time known, recent studies have also reported promising anticancer effects in various human cancers *in vitro* and *in vivo* [294]. In a research from Gao and coworkers, Apigenin was found to potentiate the cytotoxicity of Doxorubicin in HCC-derived BEL-7402/ADM cells that are otherwise resistant. Mechanistically, Apigenin strongly reduced both NRF2 mRNA and protein levels through downregulation of the PI3K/AKT pathway, leading to a reduced expression of antioxidant genes. Of note, in BEL-7402 xenografts, the coadministration of both the drugs produced synergistic effects through the inhibition of tumor growth and the induction of apoptosis [265] (see Table 1).

*(7) The Diterpenoid Oridonin*. Another natural compound, Oridonin, a bioactive diterpenoid isolated from *Rabdosia rubescens*, has been proved to possess potent anticancer effects in solid and hematologic tumors [295–297] (see Table 1). In a recent work form the group of Lu, Oridonin was seen to decrease cell viability of several osteosarcoma cell lines triggering ROS generation and apoptotic cell death. Mechanistically, these effects were found to be caused by Oridonin-dependent inhibition of the NRF2 and NF-*κ*B nuclear translocation and subsequent activation, an event associated to decreased *HO-1* and *NQO1* expression and cell death induction. Moreover, the anticancer effects of Oridonin were also subsequently confirmed *in vivo* using a xenograft tumor model [298] (see Table 1), suggesting that Oridonin might represent a promising anticancer agent given that its ability to alter the redox homeostasis of malignant cells might in turn potentiate the cytotoxicity of other prooxidizing drugs in different types of tumors.

*(8) The Cardenolide Glycoside Convallatoxin*. Other research has investigated the role of Convallatoxin, a cardenolide glycoside extracted from *Convallaria majalis* and the trunk bark of *Antiaris toxicaria*, known for acting as a Na+/K+-ATPase inhibitor but recently reconsidered in cancer research due to its ability of inducing autophagic and apoptotic cell death in several cancer cell lines [299]. Importantly, from a screening of 644 natural compounds, Convallatoxin emerged as a novel and potent NRF2 inhibitor, presumably by promoting GSK-3*β*/*β*-TrCP-dependent but KEAP1-independent proteolysis of NRF2. Notably, Convallatoxin sensitized A549 cells to 5FU-induced apoptosis, providing evidence that this natural compound might be a promising chemotherapeutic adjuvant in NSCLC treatment [300] (see Table 1).

*(9) The Lignan Honokiol*. Also, Honokiol, a lignan isolated from the bark, seed cones, and leaves of trees from the genus *Magnolia*, was reported to induce prominent toxicity in lymphoid malignant Raji and Molt4 cell lines. Mechanistically, Honokiol markedly activated the JNK pathway while in contrast, it strongly reduced both NF-*κ*B activity and NRF2 protein levels, leading to increased ROS production and apoptosis, as further confirmed in BALB/C nude mice injected with Raji cells. Thus, also these data suggest that blocking the NRF2 antioxidant response might effectively induce apoptosis also in lymphoid malignant cells and therefore should be considered a promising strategy in the treatment of nonsolid tumors [301] (see Table 1).

*(10) The Febrifugine Derivative Halofuginone*. Another promising compound with antineoplastic activity is the quinazoline alkaloid Halofuginone, a synthetic derivative of febrifugine. Despite that its inhibitory action has been initially ascribed to the synthesis of collagen type-I [302] and prolyl-tRNA synthetase [303], recent data indicate that Halofuginone can also indirectly inhibit NRF2 in therapy-resistant cancer cells with constitutive NRF2 activation [304]. Here, Halofuginone was shown to induce the amino acid starvation response due to prolyl-tRNA synthetase blockage and global protein synthesis inhibition. As a consequence of its short half-life, NRF2 was rapidly depleted even in the absence of KEAP1-mediated degradation and could not accumulate in the cytosol. Interestingly, not only Halofuginone was shown not only to decrease the proliferation of NSCLC-derived A549 and ESCC-derived KYSE70 cells with constitutive NRF2 activation, but also to enhance the efficacy of common anticancer drugs such as Cisplatin and Doxorubicin both *in vitro* and *in vivo [*304*].* Taken together, these data support the notion that Halofuginone might represent a valid chemosensitizing agent, especially in NRF2-addicted tumors.

*(11) The Naphthoquinone Plumbagin*. Also, Plumbagin, a naphthoquinone with known anticancer effects isolated from the root of the medicinal plant *Plumbago zeylanica*, was recently shown to interfere with the mitochondrial electron transport chain downstream complex II, promoting oxidative stress-dependent increase of the NRF2 activity in several human cancer cell lines. Importantly, the combined use with Brusatol displayed synergistic effects in decreasing cell proliferation and viability, suggesting that Plumbagin analogs with safe toxicity profiles might be coupled with NRF2 inhibitors for therapeutic purposes in several cancers. These observations indicate that the redox imbalance caused by Plumbagin might be exploited to induce ROS-dependent cell death in cancer cells wherein the antioxidant response triggered by NRF2 signaling is concurrently impaired [305].

*(12) The Alkaloid Berberine*. Other work has investigated the role of Berberine, a natural alkaloid abundantly present in the roots, rhizomes, stems, and bark of several medicinal plants. Known for its anti-inflammatory, antimicrobial, and antihelminthic effects [306], Berberine was recently found to exert also antineoplastic activity in breast cancer by inducing oxidative stress [307, 308]. With this respect, Zhang and coworkers have focused on BT-474 and AU-565 breast cancer cells resistant to Lapatinib, a novel tyrosine kinase inhibitor of HER2/EGFR (epidermal growth factor receptor), used to treat *HER2*-positive breast cancer. Here, Berberine was found to induce apoptosis of Lapatinib-resistant cells by reversing the c-MYC- and GSK-3*β*-dependent activation of the NRF2 antioxidant response, leading to ROS accumulation (see Table 1). Thus, the authors proposed that Berberine might be used in a combinatorial regimen to overcome Lapatinib resistance in breast cancer patients [309]. Nevertheless, additional studies need to clarify whether Berberine might also possess some therapeutic efficacy in additional types of tumors.

*(13) The Sesquiterpene Parthenolide*. Other studies focused on Parthenolide, a natural sesquiterpene lactone abundantly present in medicinal plants (especially feverfew), known for its anti-inflammatory and anticancer properties based on ROS modulation [310–313]. Recent data indicate that Parthenolide (PN) and its soluble analog dimethylamino Parthenolide (DMPN) can suppress mammosphere formation in triple-negative breast cancer (TNBC) cell lines and decrease the viability of mammosphere-derived CSC, by promoting NRF2 downregulation and increased ROS production, presumably by enhancing its ubiquitination and proteasomal degradation. Thus, it has been proposed that both PN and DMPN could be used in association with other drugs including platinum agents or with radiotherapy to increase oxidative stress and cytotoxicity in CSC from TNBC [314] (see Table 1), as previously observed in prostate cancer cells [315, 316] (see Table 1).

*(14) The Flavonoid Wogonin*. Lastly, another promising compound for cancer treatment and prevention has been Wogonin, a flavonoid isolated from the root of *Scutellaria baicalensis Georgi*. With this respect, Zhong et al. reported that breast cancer cells resistant to Doxycycline (MCF-7/DOX) were characterized by higher expression of *NRF2* and higher content of the antioxidant enzymes HO-1 and NQO1 compared to sensitive MCF-7 cells. Importantly, the resistant phenotype of MCF-7/DOX cells could be partially reversed by treatment with Wogonin, inducing a decrease in the NRF2 nuclear content, events phenocopied also by the use of NRF2 siRNAs [317] (see Table 1). The same group subsequently confirmed that in HepG2 cells, Wogonin prevented the NRF2 nuclear translocation, promoting ROS-dependent cell death and increased susceptibility to common anticancer drugs, by also reducing the activity of MRPs [318] (see Table 1). More recently, Wogonin was found to selectively induce cell death in HNC cells, sparing normal cells, and to sensitize resistant HNC cell lines (AMC-HN4R and -HN9R) to Cisplatin both *in vitro* and *in vivo* by promoting increased ROS accumulation. Mechanistically, Wogonin was seen to impair the NRF2-dependent antioxidant defense and to induce the activation of cell death pathways involving PUMA (p53-upregulated modulator of apoptosis) and PARP (poly ADP ribose polymerase). Therefore, the authors speculated that Wogonin might be a useful agent to overcome Cisplatin resistance in HNC [319] (see Table 1). Of note, Wogonin has also been reported to exert antitumor activity in two recent studies focusing on hematologic malignancies. In this regard, Xu et al. used a model of human-resistant CML (chronic myeloid leukemia) to demonstrate that Wogonin could reverse the phenotype of Adriamycin- (ADR-) resistant human myelogenous leukemia K562/A02 cells. Here, the inhibition of the PI3K/AKT pathway was found to decrease in turn the *NRF2* mRNA levels, causing suppression of MRP1 activity and expression and reducing the protein content of both HO-1 and NQO1 [320] (see Table 1). On the other hand, the same group further clarified the mechanism by which Wogonin suppressed *NRF2* transcription in resistant K562/A02 CML cells, providing evidence that the functional inactivation of NF-*κ*B was fully responsible for the inhibition of the NRF2/ARE pathway. Moreover, when combined with Adriamycin, Wogonin potentiated the inhibitory effect of ADR on tumor growth in NOD/SCID mice xenografted with K562/A02 CML cells, by suppressing the STAT3/NF-*κ*B/NRF2 pathway [321] (see Table 1). Collectively, these studies strongly support the notion that Wogonin or its other derivatives can represent potent chemosensitizers in different types of solid and hematologic tumors with intrinsic or acquired resistance to therapy. Importantly, since Wogonin has been shown to induce anti-inflammatory and chondroprotective effects through the activation of ROS/ERK/NRF2 pathways in human osteoarthritis chondrocytes [322], it is possible that this compound might regulate the NRF2 function in two opposite directions depending on the context of normal or cancer cells. If confirmed by other studies, this property might select Wogonin or its derivative as specific anticancer agents that might selectively induce effective killing of malignant cells sparing the normal nontransformed cells.

*(15) Other Promising Natural Compounds*. Intriguingly, it has also been reported that certain natural compounds can exert antineoplastic activities despite promoting paradoxical activation of NRF2, suggesting that the specific context might ultimately dictate the outcome of NRF2 modulation. For example, the polyphenol EGCG (epigallocatechin gallate) has been shown to induce chemosensitization to Cisplatin in TNBC MDA-MB231 cells and to suppress tumor growth in xenografted mice, by inducing an NRF2-dependent antioxidant response with minimal side toxicity on normal cells. Therefore, this indicates that NRF2 activators can also synergistically enhance the efficacy of common anticancer agents [323]. In another study, the phytochemical mollugin, a bioactive compound with known antitumor activity isolated from *Rubia cordifolia L.* (Rubiaceae), was found to induce cell death in primary and metastatic OSCCs (oral squamous cell carcinoma). Mechanistically mollugin was found to suppress NF-*κ*B downstream signaling and the expression of both antiapoptotic and proangiogenic genes and also to induce NRF2-dependent *HO-1* expression due to p38, ERK, and JNK pathway activation [324]. Intriguingly, some natural compounds were also seen to effectively induce cancer cell death despite promoting a paradoxical activation of NRF2. For example, dehydroepiandrosterone (DHEA), an endogenous hormone with anticancer properties, was found to promote autophagic cell death in HepG2 cells through the ROS-independent activation of JNK which in turn elicited NRF2 nuclear translocation and promoted p62 expression to induce autophagy. Thus, it has been proposed that DHEA might represent an appealing drug for killing cancer cells refractory to apoptosis by triggering p62-dependent autophagic cell death [325].

Taken together, these data demonstrate that natural compounds and their derivatives, by virtue of their prooxidizing ability, might be promising anticancer agents in different clinicopathological settings, especially in those tumors that strongly rely on NRF2-dependent antioxidant functions to cope with oxidative stress induced by alterations in the microenvironment or the administration of anticancer drugs.

#### 4.1.3. Interfering with Oncogenic Functional Interactors of the NRF2/KEAP1 Pathway

*(1) Inhibitors of PI3K, DNA-PK, and ERK*. It is well established that many upstream regulators and downstream effectors can influence the activation status of and the biological effects exerted by the NRF2/KEAP1 pathway. Despite that the list of this functional interactors is continuously expanding, in this section, we will describe some of the most relevant oncogenic signaling pathways that converge on NRF2 activation. It should be emphasized that the pharmacologic inhibition of these molecular targets has been largely exploited for drug repurposing, providing encouraging results in the context of therapy-resistant tumors. With this respect, by using human pancreatic cancer cell lines and a xenograft model, the PI3K/DNA-PK inhibitor known as PIK-75 was found to decrease the NRF2 protein levels and its transcriptional activity by proteasome-mediated degradation. The first-line treatment for PDAC is represented by Gemcitabine, but a large proportion of treated patients becomes refractory, in part due to the upregulation of the NRF2 activity. Importantly, when used as an adjuvant, PIK-75 was able to counteract the increase in NRF2 induced by Gemcitabine and to significantly potentiate its antitumor effects both *in vitro* and *in vivo* [326]. Notably, this study provides a strong mechanistic rationale to employ NRF2-targeting agents in combination with Gemcitabine for improving the clinical outcome of patients affected by otherwise resistant PDAC. In another study conducted on U251 human glioblastoma cells, the ERK and PI3K signaling cascades were found to regulate the expression and activation of NRF2, while the cotreatment with pharmacologic inhibitors (PD98059 for ERK and LY292004 for PI3K) was able to revert these changes and trigger cell death [327]. Interestingly, LGB-321 and AZD1208, two inhibitors of PIM kinase, a protein frequently overexpressed in many tumors exposed to hypoxia, were found to impair tumor growth and selectively kill different types of hypoxic cancer cells *in vitro* and *in vivo*, preventing NRF2 nuclear accumulation and leading to the buildup of ROS [328], suggesting that this strategy might overcome the hypoxia-mediated therapy resistance frequently encountered in the treatment of many tumors.

*(2) Inhibitors of the JNK, ERK, EGFR, and PDGFR Signaling*. Another relevant clinical problem in cancer treatment is the resistance to EGFR-TKIs (tyrosine kinase inhibitors) in NSCLC patients that are initially good responders. With this respect, by establishing Gefitinib-resistant (GR) NSCLC cells, it has been shown that the increased overexpression of several NRF2-dependent target genes was due to an acquired *KEAP1* mutation, an event promoting a malignant phenotype and cross-resistance to the EGFR-TKIs Afatinib and Osimertinib both *in vitro* and *in vivo*. Here, the inhibition of NRF2, either by treatment with Brusatol or by restored expression of wild-type *KEAP1*, suppressed tumor cell proliferation and tumorigenicity *in vitro* and *in vivo*, confirming that deregulation of the NRF2/KEAP1 pathway can be responsible for the acquired resistance to EGFR-TKIs observed in many NSCLC patients, while its pharmacologic ablation might represent a valid option to overcome this phenomenon [329]. Notably, in the same context, the group of Zhong confirmed that NRF2 activation contributed to the resistance of NSCLC to EGFR-TKI treatment in wild-type EGFR NSCLC cells. Here, the authors demonstrated that Icotinib and Gefitinib triggered apoptosis in EGFR mutant HCC827 but not in EGFR wild-type A549 NSCLC cells without inducing protective autophagy. Moreover, suppression of the NRF2 activity with the inhibitor Brusatol significantly reduced the cell survival of A549 cells, without further sensitizing them to EGFR TKI-induced cell death, suggesting that suppression of NRF2 can be used to induce autophagy-independent cell death in NSCLC tumors [330]. In another work, adenoviral transduction was used to express both melanoma differentiation-associated gene-7 (*MDA-7*) and interleukin-24 (*IL-24*), two known inducers of apoptosis by ROS increase, in different cancer cell lines. The adenovirus ZD55-IL-24 promoted the association between NRF2 and KEAP1 and attenuated the ARE-dependent gene transcription by activating the p38/JNK but inhibiting the ERK pathways in A549, leading to tumor-specific apoptosis [331]. Also, in the attempt of finding novel therapeutic combinations against TNBC, Ebelt et al. explored the potential use of the EGFR inhibitor Lapatinib and the c-Jun N-terminal kinase inhibitor JNK-IN-8. Surprisingly, the synergistic combination of the drugs was found to decrease the transcriptional activity of NRF2, inducing oxidative stress-dependent cell death in MDA-MB-231 and MDA-MB-436 breast cancer cells by strongly depleting the intracellular levels of GSH and NADPH, observations further confirmed in human xenograft tumors [332]. Lastly, an unexpected implication for NRF2 emerged from a recent work wherein the combined use of an HER2 inhibitor, Trastuzumab with an EGFR-inhibitor, Nimotuzumab, was assessed in the context of *HER2*-overexpressing breast cancer. In this case, the greater antitumor activity exerted by the drug combination was found to be at least in part dependent on the ROS generation due to repression of the NRF2 pathway [333]. Another recent study investigated the anticancer effects of CP-673451, a selective PDGFR*β* inhibitor, in models of NSCLC. Here, CP-673451 was found to suppress *NRF2* expression and promote a significant increase in cell apoptosis, accompanied by ROS accumulation in A549 and H358 NSCLC cell lines that was further exacerbated by the coadministration of Cisplatin [334]. Taken together, these data support the concept that NRF2 is a crucial determinant of therapy resistance against TKRIs in lung and breast cancers and further highlight the importance of combinatorial regimens wherein NRF2 inhibition might represent the prerequisite to restore or at least improve the efficacy of already established drugs and achieve optimal therapeutic effects.

*(3) Other Inhibitors*. Of note, interesting observations were obtained in a model of head and neck cancer (HNC) wherein the authors identified a combinatorial treatment to overcome the resistance mechanism to the small-molecule RITA (reactivation of p53 and induction of tumor cell apoptosis), an inducer of p53-independent apoptosis. Here, different RITA-resistant HNC cell lines with sustained activation of both autophagy- and NRF2-dependent antioxidant pathways were found to display an increased sensitivity to RITA in the presence of the autophagy inhibitor 3-MA, while the same combination of drugs was able to increase oxidative stress and DNA damage in HNC cells xenografted into recipient mouse models. Mechanistically, p62 downregulation was found to mediate suppression of the NRF2/KEAP1 pathway, promoting KEAP1 accumulation and NRF2 degradation, paralleled by decreased content of the ARE-regulated antioxidant enzymes GCLC, GCLM, HO-1, and NQO-1 [335]. In another context, HepG2 cells treated with the WNT3A (Wingless/int-3A) inhibitor LGK-974 showed a marked decrease in the proliferation rate and became more sensitive to radiation-induced apoptosis, due to the inhibition of both the WNT and NRF2 signaling. Here, the authors proposed that protein complexes formed by WNT and AXIN1/GSK-3*β* could interact with NRF2 in the cytosol and prevent its nuclear translocation, favoring instead *β*-TrCP-mediated NRF2 proteasomal degradation [336]. Therefore, it seems that a functional interaction between WNT and NRF2 might be responsible of radioresistance in HCC while the canonical WNT inhibitor LGK-974 might serve as a radiosensitizing drug in those types of tumors wherein this protective mechanism is activated. Once again, however, it should be noted that targeting upstream regulators of the NRF2 protein is a valuable therapeutic strategy to interfere with its prooncogenic function and induce ROS-dependent cytotoxicity.

#### 4.1.4. RNAi against NRF2 or Its Regulators

Apart from the use of chemical compounds with inhibitory effects, genetic inactivation of *NRF2* by RNA interference is also a promising strategy to selectively impair NRF2 activity and overcome chemoresistance. With this respect, the role of NRF2 in tumor growth and Docetaxel sensitivity was investigated in *ErbB2*-overexpressing ovarian carcinoma SKOV3 cells, wherein the stable NRF2 depletion by RNAi was able to repress NRF2-dependent signaling, leading to cell growth arrest and tumor growth retardation in mouse xenografts. Of note, the *ErbB2* expression was significantly reduced in NRF2-inhibited SKOV3 cells, whose sensitivity to Docetaxel was in turn increased. The same effect was confirmed also in the *ErbB2*-positive breast cancer cell line BT-474 [337], supporting the notion that NRF2 inhibition with RNAi might be a therapeutic strategy to limit tumor growth and enhance sensitivity to taxane-based chemotherapy. In another study, MDA-MB-231 breast carcinoma cells with stable *NRF2* knockdown displayed enhanced sensitivity to photodynamic therapy (PDT) due to increased ROS levels. Importantly, these observations were also confirmed in breast MCF-7, colon HCT116, renal A498 carcinoma, and glioblastoma A172 cells, indicating that genetic ablation of *NRF2* might potentially increase the efficacy of PDT in malignant tumors of different origin by altered redox homeostasis and cytotoxic ROS accumulation [338]. Also, a long intergenic noncoding RNA (lincRNA) named AATBC, overexpressed in bladder cancer patient tissues, was found to promote an aggressive phenotype and was associated to poor prognosis. Interestingly, knockdown of *AATBCC* by siRNAs was able to downregulate NRF2 protein levels and increase the sensitivity of UM-UC-3 and EJ bladder cancer cells to Cisplatin [339]. Also, it has been proposed that the use of NRF2 siRNAs might have a therapeutic relevance in the treatment of laryngeal squamous cancer, since Hep-2 cells refractory to Cisplatin due to high levels of the antioxidant enzyme HO-1 were found to be strongly sensitized by *NRF2* knockdown and subsequent ROS elevation [340]. Lastly, siRNAs against *NRF2* were found to enhance the cytotoxicity of Cisplatin in human cholangiocarcinoma KKU-100 cells, further elevating the production of ROS normally induced by the single administration of various anticancer drugs [341]. Collectively, this evidence confirms that siRNA-mediated targeting of the NRF2 pathway holds a great therapeutic potential and will presumably be the focus of extensive studies in the near future. Despite that additional work will be necessary to improve the specificity and efficiency of siRNA delivery into tumor cells and minimize the nonspecific stimulation of the immune response, it is expected that siRNA-based drugs will pave the way to a new and exciting era in cancer treatment.

#### 4.1.5. Positive Regulation of KEAP1 Suppressive Function

Another potential strategy to suppress NRF2 oncogenic signaling is represented by the restoration of KEAP1 negative control that might be achieved either by its functional reactivation/reintroduction or by promoting its physical interaction with NRF2. In this section, we will describe some of the most recent studies that have tried to exploit these mechanisms. In this regard, an earlier study reported that high levels of NRF2 were associated to Cisplatin and Paclitaxel resistance in endometrial serous carcinoma (ESC). Here, forced overexpression of *KEAP1* was shown to sensitize SPEC-2 cells to several chemotherapeutics both *in vitro* and in xenografted SCID mice [342], thus indicating that reintroduction of a functional KEAP1 might effectively inhibit the NRF2 activity. In another work from Leone et al., the cotreatment with Vorinostat, a histone deacetylase inhibitor (HDACI) and either of two different EGFR-TKIs (Gefitinib, Erlotinib), was shown to suppress the c-MYC-regulated NRF2 function, increasing the levels of KEAP1 and promoting oxidative stress-dependent apoptosis in NSCLC cells. These results support the notion that therapeutic manipulations of the redox homeostasis induced by KEAP1 restoration can improve the outcome of resistant NSCLC [343]. Of note, experimental approaches aimed at promoting KEAP1/NRF2 interaction have also been recently reported. In this regard, by using a HTS screening on 150000 compounds, Saito et al. identified K67, a small noncovalent inhibitor of phospho-p62/KEAP1 interaction, as a molecule capable of restoring the main route of NRF2 degradation in human HCC lines. Importantly, K67 exerted also antineoplastic effects in several HCC cell lines by decreasing proliferation and enhancing the cytotoxicity of either Sorafenib or Cisplatin, confirming that this inhibitor might be exploited to treat HCC cancers with p62-dependent NRF2 hyperactivation [216]. However, due to K67 low solubility, the authors proposed that changes in its chemical structure would have been required before performing any clinical study. Thus, in a subsequent work from the same group, novel K67 derivatives with various side chains on the C-2 naphthalene ring position were designed, despite that their pharmacologic properties and biological effects in human therapy still need to be clarified [230]. A recent study was also pursued to clarify the mechanisms behind the antitumor effects of the natural compound 2′,4′-dihydroxy-6′-methoxy-3′,5′-dimethylchalcone (DMC), a chalcone extracted from the buds of *Cleistocalyx operculatus*. Here, by using therapy-resistant HCC BEL-7402/5-FU cells, the authors showed that DMC promoted a significant increase in the KEAP1 protein levels, preventing NRF2 nuclear translocation and subsequent target gene transactivation. As a consequence, reduced expression of the *GCLC* and *GCLM* genes was found to markedly decrease the intracellular GSH content, while an elevation of the ADP/ATP ratio was found to attenuate the MRP1-mediated drug efflux, reversing the resistant phenotype [344]. Taken together, these data indicate that even in the absence of direct NRF2 inhibition, restoring the negative control exerted by KEAP1 can efficiently suppress NRF2 downstream signaling. It is however expected that this strategy might not be effective in cancer cells with compromised activity of those molecular pathways controlling NRF2 proteasomal degradation.

#### 4.1.6. Repurposed Drugs

In the last years, the phenomenon of drug repurposing, based on the use of already established therapeutics for new indications, has gained great attention, particularly in the context of cancer treatment where the development of new drugs might be time-consuming and cost prohibitive [345]. In the following paragraphs, we will describe recent studies wherein drug repurposing has led to the attenuation of NRF2 signaling, overcoming major clinical hurdles in the treatment of various malignant tumors.

*(1) Repurposing of the All-Trans Retinoic Acid (ATRA)*. Retinoic acid, an active metabolite of vitamin A, known to promote cell differentiation and inhibit proliferation has been the focus of extensive research [346]. Initial studies from Wang and coworkers reported that all-trans retinoic acid (ATRA) and other retinoic acid receptor alpha (RARalpha) agonists markedly impaired NRF2-dependent induction of ARE-driven genes by cancer chemopreventive agents but not its nuclear translocation in human breast cancer MCF-7 cells [347] (see Table 2). Subsequent investigations led to the identification of a slightly different mechanism of NRF2 inhibition in acute myeloid leukemia (AML) and acute promyelocytic leukemia (APL) cells, wherein ATRA was shown to prevent the nuclear accumulation of NRF2 in response to arsenic trioxide (ATO), enhancing its cytotoxicity due to impaired transactivation of antioxidant target genes [348] (see Table 2). Other experimental work from the group of Furfaro focused on malignant neuroblastoma, wherein the activation of NRF2 has been proposed to promote resistance to proteasome inhibitors such as Bortezomib (BTZ). Here, using the highly chemoresistant HTLA-230 neuroblastoma cells, the authors showed that BTZ treatment induced the NRF2-dependent transcription of multiple antioxidant genes (*HO-1*, *GCLM*, and *x-CT*) that conferred resistance by increasing the intracellular GSH content. Importantly, ATRA administration was found to impair the binding of NRF2 to the ARE sequences, decreasing both the *HO-1* induction and the intracellular GSH content and consequently enhancing the efficacy of low BZT doses [349] (see Table 2). Also, in a model of human glioblastoma (GBM), NRF2 was found to be implicated in the resistance to Temozolomide (TMZ), the most commonly used first-line chemotherapeutic for GBM patients. In this context, ATRA was found to significantly decrease both the mRNA and protein levels of NRF2 in U251 glioma cells and to potentiate the antitumor effects of TMZ [349]. Lastly, in a very recent work, Kim et al. investigated the potential role of NRF2 signaling in CSC-like properties of ovarian CSCs exhibiting high enzymatic activity of aldehyde dehydrogenase1 (ALDH1) and high expression levels of p62, a hallmark associated also to aggressive behavior and drug resistance. Here, ATRA was found to suppress NRF2 activation by hampering *ALDH1* and *p62* expression, leading to a marked attenuation of the CSC features of ovarian cancer cells with high ALDH1 activity. Among these features, chemoresistance, colony/sphere formation, tumor growth, and the expression of CSC markers were strongly abrogated, an effect that was also produced by *NRF2* silencing [350] (see Table 2). Taken together, these studies highlight the therapeutic potential of ATRA in the treatment of solid and nonsolid tumors with aggressive or therapy-resistant phenotypes.

*(2) Repurposing of the Cdc7/CDK9 Inhibitor PHA-767491*. In another context, by screening a collection of 5879 known bioactive compounds and FDA-approved drugs in HepG2, Liu and coworkers demonstrated that the CDC7/CDK9 inhibitor PHA-767491 was also a potent suppressor of NRF2 transcriptional activity. Validation assays performed in MM cells confirmed that PHA-767491 prevented NRF2 nuclear translocation, increased the mitochondrial superoxide generation, and suppressed cell growth [351] (see Table 2).

*(3) Repurposing of the Multi-TKI Sorafenib*. Another relevant clinical obstacle in patients with advanced HCC is represented by the acquired resistance to 5-FU. With this respect, Zhou and coworkers have shown that NRF2 is directly implicated in this phenomenon and its expression is significantly increased in Bel-7402/5-FU-resistant cells subdued to the drug treatment. Interestingly, by using these cells, the authors demonstrated that subtoxic doses of the multikinase inhibitor Sorafenib, normally used to target tumor angiogenesis, were able to reverse the drug resistance suppressing the NRF2 increase induced by 5-FU, although the mechanisms were not fully elucidated. However, this indicates that NRF2 inhibitors might effectively increase the efficacy of many chemotherapeutics in HCC patients [352] (see Table 2).

*(4) Repurposing of the TXNRD1 Inhibitor Auranofin*. Promising results were also obtained in a model of lung cancer since the thioredoxin reductase 1 (TXNRD1) inhibitor Auranofin, a known antirheumatic agent, was found to synergistically enhance the toxicity of TUSC2/Erlotinib, a treatment currently in phase II clinical trials in stage 4 NSCLC patients refractory to other options. Here, in the presence of Auranofin, several cancer cell lines exhibited increased susceptibility to the TUSC2/Erlotinib combination, undergoing massive ROS-dependent apoptosis at least in part dependent on a deficitary NRF2-mediated transcriptional response, although the detailed mechanisms were not investigated [353] (see Table 2).

*(5) Repurposing of the Corticosteroid Clobetasol Propionate*. Also, by using a cell-based assay on A549 NSCLC cells, Choi and coworkers screened almost 4000 clinical compounds, leading to the identification of the glucocorticoid clobetasol propionate (CP), a drug used to treat dermatologic disorders, as a potent NRF2 inhibitor. Mechanistically, CP was found to prevent NRF2 nuclear accumulation and promote its degradation through the *β*-TrCP-dependent pathway, leading to ROS accumulation and marked suppression of anchorage-independent growth in several NSCLC cell lines with mutant *KEAP1*. Moreover, when used alone or in combination with Rapamycin *in vitro* or *in vivo*, CP impaired the growth of tumors harboring *KEAP1* or both *KEAP1*/*LKB1* mutations, a frequent event in lung cancer. Therefore, CP could be a repurposed therapeutic agent for tumors with high NRF2 activity while the combined use of CP and Rapamycin might be a valid clinical approach in tumors with *KEAP1* and *LKB1* mutations [354] (see Table 2).

*(6) Repurposing of the Topoisomerase Inhibitor Camptothecin*. Another clinical obstacle that has taken advantage of repurposing strategies is the chemoresistance of HCC. With this respect, in the attempt to find new molecules targeting NRF2, Chen et al. have shown that the topoisomerase inhibitor Camptothecin could markedly suppress *NRF2* expression and downstream target gene transactivation in different cancer cell lines such as HepG2, SMMC-7721, and A549. Despite the lack of more precise mechanistic explanation, the authors proposed that the inhibitory effect of CPT on *NRF2* expression might be related to suppression of its transcription, translation, or even promotion of its mRNA degradation. Also, Camptothecin at micromolar doses was found to sensitize these cells to a great variety of anticancer drugs *in vitro* and *in vivo*, suggesting that this drug might be repurposed to effectively treat malignant tumors with high basal *NRF2* expression [23] (see Table 2).

*(7) Repurposing of the Histone Deacetylase Inhibitor Valproic Acid*. Interestingly, in a recent work, the tumor necrosis factor-related apoptosis-inducing ligand (TRAIL), an effective agent for the treatment of many cancers, was found to synergize with the histone deacetylase inhibitor, valproic acid (VPA), in models of human papillary thyroid cancer (PTC) both *in vitro* and *in vivo*. Mechanistically, the TRAIL-VPA combination increased the apoptotic rate of TRAIL-resistant PTC cells by downregulating the NRF2-dependent antioxidant response, decreasing its nuclear accumulation presumably due to reduced *Notch1* expression; these effects were further enhanced when siRNAs against these proteins were combined with TRAIL or TRAIL-VPA treatments. Therefore, the concomitant use of VPA and TRAIL might constitute a promising therapy for TRAIL-resistant PTC and a powerful combination to promote cell death [355] (see Table 2).

*(8) Repurposing of the Antidiabetic Biguanide Metformin*. Another molecule that has received great attention in drug repurposing for cancer treatment is Metformin, a biguanide commonly used to treat type II diabetes. In this regard, earlier studies reported that Metformin inhibited proliferation in several cancer cell lines by suppressing *HO-1* expression through the inhibition of a RAF/ERK/NRF2 signaling and AMPK-independent pathways that promoted a marked decrease in the NRF2 protein content [356]. Subsequently, the same group, using human cancer cell lines expressing different forms of p53, demonstrated that Metformin was able to induce miR-34a and to suppress the SIRT1/PGC-1*α*/PPAR*γ*-mediated transcription of the *NRF2* gene, leading to decreased expression of *SOD2* and *HMOX1* and augmented sensitivity of wild-type p53 cancer cells to oxidative stress [357] (see Table 2). In another study focused on the mechanisms underlying progestin resistance in endometrial precancer/cancer, the antioxidant NRF2/AKR1C1 pathway was found to be hyperactivated in progestin-resistant endometrial epithelia, but not in responsive endometrial glands. Here, Metformin or Brusatol administration was found to overcome progestin resistance by downregulating *NRF2* and *AKR1C1* expression, an effect produced also by *NRF2* or *AKR1C1* silencing. Therefore, the authors proposed that downregulation of NRF2 and AKR1C1 through Brusatol or Metformin administration might be useful to overcome progestin therapy failure in patients with endometrial cancer that require a more conservative approach [358] (see Table 2). In a similar context, very recent data from Bai and coworkers demonstrated that the enzyme isocitrate dehydrogenase 1 (IDH1) was highly expressed and aberrantly activated in endometrial cancer tissues and lines, promoting chemoresistance. Mechanistic studies revealed the existence of a feedback loop involving NRF2 through which the IDH1-derived *α*-KG positively modulated the activity of the dioxygenase TET1, which in turn enhanced the conversion of 5-methylcytosine (5mC) to 5-hydroxymethylcytosine (5hmC) within the *NRF2* promoter region, leading to the expression of target genes, including *IDH1* itself. Importantly, Metformin was shown to disrupt this regulatory loop repressing the IDH1/*α*-KG-dependent activation of TET1 and attenuating the hydroxymethylation levels of the *NRF2* promoter, ultimately restoring the chemosensitivity of various endometrial cancer cells [359] (see Table 2). Encouraging evidence was also obtained in a model of human colon carcinoma (CRC) in a recent study from Sena and coworkers. Here, Metformin was shown to exert antineoplastic effects by inhibiting cell proliferation and enhancing apoptotic cell death in HT29 CRC cell lines, as a consequence of the transcriptional inactivation produced on NRF2 and NF-*κ*B [360] (see Table 2). Also, additional research from Yu and coworkers demonstrated that Metformin was able to sensitize A549 NSCLC cells but not normal lung epithelial BEAS-2B cells, to the natural compound EGCG by inducing ROS-dependent apoptosis. Mechanistically, Metformin upregulated *SIRT1* expression through the NF-*κ*B pathway decreasing NRF2 acetylation and nuclear translocation, leading to reduced expression of *HO-1*. Importantly, either Metformin or EGCG inhibited the tumor growth of NSCLC A549 xenografted in BALB/c nude mice, showing addictive effects when used in combination [361] (see Table 2). Lastly, Metformin was shown to suppress *HO-1* mRNA and protein expression in human HCC HepG2, cervical cancer HeLa, and NSCLC A549 cells, markedly reducing *NRF2* mRNA and protein levels by inactivating RAF/ERK signaling [356] (see Table 2).

*(9) Repurposing of the Antitubercular Agent Isoniazid*. Interestingly, the antitubercular agent Isoniazid (INH) known to induce hepatotoxicity in patients subdued to long-term treatment was found to induce ROS accumulation and apoptosis in HepG2 HCC and in transformed human liver THLE-2 cells, by preventing NRF2 nuclear translocation due to the inhibition of its importer Karyopherin *β*1 [362] (see Table 2). Taken together, these studies indicate that Metformin, an already widely used antidiabetic drug with a safe toxicity profile and multiple molecular targets, might be a promising drug for the treatment or the prevention of various cancers while INH might be successfully employed in HCC to evoke ROS-dependent cytotoxicity.

## 5. Future Perspectives and Conclusions

It is becoming increasingly clear that NRF2 plays a crucial role in cancer malignancy and therapy resistance by controlling the intracellular redox homeostasis through the activation of cytoprotective antioxidant genes. A growing number of studies suggest that suppression of NRF2-related antioxidant mechanisms might represent a feasible and promising therapeutic approach to induce prooxidizing conditions in the tumor microenvironment and trigger ROS-dependent cell death in several human malignancies. Despite the lack of specific and selective NRF2 inhibitors, compelling evidence indicates that the use of natural compounds or even the repurposing of preexisting drugs with known pharmacokinetic and toxicity profiles might be successfully employed as single agents or chemosensitizing adjuvants in different types of tumors, thus encouraging further investigations [269, 341, 363, 364]. Of note, given the functional location of NRF2 at the crossroad of multiple pathways, pharmacologic manipulations of upstream regulators or downstream effectors of NRF2 signaling might also produce remarkable anticancer effects and synergize with already established drugs through mechanisms that almost invariantly converge on the disruption of the intracellular redox homeostasis [131, 363, 365, 366]. Therefore, it is expected that in the near future, additional studies will explore other favorable combinations to hamper the prooncogenic functions of NRF2 and its biological effects, with the aim of discovering novel and effective therapeutic alternatives against cancers with otherwise limited options and NRF2 addiction. On the other hand, it should also be noted that the discovery of novel NRF2/ARE inhibitors with sufficient potency, specificity, and safety profiles still represents a critical challenge in the field of cancer research that might lead to a breakthrough in the strenuous fight against cancer.

## Figures and Tables

**Table 1:** 

Compound	Target	Tumor type/cell lines	Effect	Ref no.
AEM1	Sirt2, NRF2	NSCLC/A549	Decreases NRF2 transcriptional activity	[269]
ML385	Neh1 domain of NRF2	NSCLC/A549	Impairs the DNA interaction of the MAFG-NRF2 complex	[25]
Procyanidins from CCE	IGF1R	NSCLC/A549	Promotes proteasome-independent NRF2 degradation through IGF1R phosphorylation	[271–273]
Luteolin	NRF2	NSCLC/A549	Decreases NRF2 mRNA and protein levels	[274, 275]
Trigonelline	NRF2 import system	PDAC/Panc-1, MiaPaca2; PAC/Colo357	Decreases the nuclear levels of NRF2	[276]
Brusatol	Overall protein translation	NSCLC/A549; TNBC/MDA-MB-231; PDAC/Panc1; PAC/BxPC3, PATU-8988; CRC/HCT116; melanoma/A537; AML/THP1, HL60	Promotes NRF2 degradation	[17, 236, 282–285, 287, 291, 292]
Chrysin	Hexokinase2, ERK1/2, NF-*κ*B, NRF2	HCC/Bel-7402ADM; glioblastoma/U87	Decreases NRF2 mRNA and protein content; decreases NRF2 nuclear translocation	[293, 294]
Apigenin	PI3K/Akt	HCC/Bel-7402ADM	Decreases NRF2 mRNA and protein content	[266]
Oridonin	PPAR*γ*, NF-*κ*B, NRF2, JNK	Osteosarcoma/MG-63, HOS; BC/MDA-MB-231, MCF7; CRC/Hct116; DLBCL/SUDHL2 and 4, OCl-Ly-3 and 8	Decreases NRF2 nuclear translocation	[296–299]
Convallatoxin	NRF2	NSCLC/A549	Promotes GSK-3*β*/*β*-TrCP-dependent NRF2 degradation	[301]
Honokiol	NF-*κ*B, NRF2	Burkitt's lymphoma/Raji; T-all/MOLT-4	Decreases NRF2 expression	[302]
Berberine	c-Myc, NRF2	BAC/BT-474, AU-565	Promotes GSK-3*β*/*β*-TrCP-dependent NRF2 degradation	[307]
Parthenolide	NF-*κ*B, JNK, STAT	TNBC/MDA-MB-231; BC/MCF7	Decreases NRF2 expression	[310–314]
Wogonin	MAPKs, NF-*κ*B, p53, cMyc, PI3K/Akt, DNA-PKcs, STAT3, NRF2	BC/MCF-7; HCC/HepG2; CML/K562-A02; HNC/AMC-HN4R and -HN9R	Decreases NRF2 content at the transcriptional level; increases KEAP1 levels	[315–319]

**Table 2:** 

Drug	Previous use	Tumor type/cell lines	Effect	Ref.
ATRA, RAR-*α* agonists	APL treatment; neuroblastoma treatment; skin disorders	BC/MCF-7; AML/HL60, THP-1; APL/NB4, NB4-R2; NB/HTLA-230; GBM/U251; OC/A2780 CSC	Decreases NRF2 binding to ARE sites; decreases NRF2 nuclear translocation	[347–350]
PHA-767491	Cdc7/CDK9 inhibitor	PDAC/PANC-1, Capan-1; HCC/HepG2	Decreases NRF2 nuclear translocation and activity	[351]
Sorafenib	Multi-Tyrosine kinase inhibitor; antiangiogenic therapy	CRC/DLD-1, HCT116; TC/FTC133, BC-PAP, 8505C; RC/ACHN, 786-O; CRC/DLD-1; HCC/HepG2, BEL7402-5FU, HuH-7; BC/MCF7, MDA-MB-231; NSCLC/CALU-3	Decreases NRF2 expression and nuclear translocation	[352]
Auranofin	Rheumatoid arthritis	NSCLC/Calu-3, Calu-6, H522	Decreases NRF2 activation	[353]
Clobetasol Propionate	Skin disorders	NSCLC/A549, H2228	Decreases NRF2 nuclear accumulation and promotes *β*-TrCP-dependent NRF2 degradation	[354]
Camptothecin	Topoisomerase inhibitor; chemotherapy	HCC/HepG2, SMMC-7721; NSCLC/A549	Decreases NRF2 expression	[23]
Valproic acid	Histone deacetylase inhibitor; epilepsy and seizure disorders; chemosensitizer	BC/MCF7; TC/BCPAP, TCP1, BHP10-3	Decreases NRF2 nuclear content	[355]
Metformin	Antidiabetic drug	BC/MCF-7; CRC/HT-29; EC/RL95–2, Spec-2, Ishikawa; HCC/HepG2; NSCLC/A549; CC/HeLa	Decreases NRF2 mRNA and protein content; decreases NRF2 expression	[356–361]
Isoniazid	Antitubercular agent	HCC/HepG2	Decreases NRF2 nuclear translocation	[362]

## Abbreviations

ROS: Reactive oxygen species
CNC: Cap“n”Collar
MAF: V-Maf avian musculoaponeurotic fibrosarcoma oncogene homolog
NTD: N-Terminal domain
BTB: Bric-à-brac
IVR: Intervening region
CTD: C-Terminal domain
CUL-1: Cullin 1
CUL-3: Cullin 3
RBX1: Ring box 1
*β*-TrCP: *β*-Transducin repeat-containing protein
HRD1: HMG-coA reductase degradation 1
ER: Endoplasmic reticulum
ARE: Antioxidant responsive elements
EpRE: Electrophilic responsive elements
GSK-3*β*: Glycogen synthase kinase-3 beta
HNSC: Head-neck squamous cell carcinoma
NSCLC: Non-small-cell lung cancer
PRCC: Papillary renal cell carcinoma
TCGA: The Cancer Genome Atlas
CNV: Copy number variations
SNP: Single-nucleotide polymorphism
MBD1: Methyl-CpG-binding domain protein 1
UHFR1: Ubiquitin like with PHD and ring finger domains 1
PDAC: Pancreatic ductal adeno carcinoma
EZH2: Enhancer of Zeste 2 polycomb repressive complex 2 subunit
BRD4: Bromodomain protein 4
HMOX1: Heme oxygenase 1 (gene)
UTRs: Untranslated regions
AML: Acute myeloid leukemia
HCC: Hepatocellular cancer
5-FU: 5-Fluorouracil
GSH: Reduced glutathione
MSA: Methylseleninic acid
ESCC: Esophageal squamous cell carcinoma
GCLM: Glutathione cysteine ligase modulatory subunit
NQO1: NAD(P)H quinone dehydrogenase 1 (gene)
ERMP1: Endoplasmic reticulum metalloprotease 1
HIF1: Hypoxia-inducible factor 1
CDKi: Cyclin-dependent kinase inhibitor
PDGFA: Platelet-derived growth factor a
PDGFR*β*: Platelet-derived growth factor receptor *β*
PALB2: Partner and localizer of BRCA2
BRCA2: Breast cancer type 2 susceptibility protein
SQSTM1: Sequestosome 1
KIR: KEAP1 interaction region
HER2: Human epidermal growth factor receptor 2
MMTV: Mouse mammary tumor virus
CSC: Cancer stem cell
FH: Fumarate hydratase
TCA: Tricarboxylic acid
HO-1: Heme oxygenase 1 (protein)
CRISPR/Cas9: Clustered regularly interspaced short palindromic repeats/Cas9
MAPK: Mitogen-activated protein kinase
MEK: MAPK/ERK kinase extracellular signal-regulated kinases
ERK: Extracellular signal-regulated Kinases
HATs: Histone acetyl transferases
HDACI: Histone deacetylase inhibitor
KRAS: Kirsten rat sarcoma viral oncogene homologue
HRAS: Harvey rat sarcoma viral oncogene homologue
PI3K: Phosphatidyl inositol-4,5-bisphosphate 3-kinase
AMPK: AMP-activated protein kinase
AICAR: 5-Aminoimidazole-4-carboxamide ribonucleotide
MMP9: Matrix metalloprotease 9
MDR: Multidrug resistance
UPR: Unfolded protein response
PERK: Protein kinase RNA-like endoplasmic reticulum kinase
MRP1: Multidrug resistance-associated protein 1
GRP78: Glucose-regulated protein of 78 kD
FGF19: Fibroblast growth factor 19
FGFR4: Fibroblast growth factor receptor 4
IGF1R: Insulin-like growth factor 1 receptor
MM: Multiple myeloma
PI: Proteasome inhibitor
CHOP: C/EBP homologous protein
MDR1: Multidrug resistance 1 gene, multidrug resistance protein 1
MRP1-5: Multidrug resistance-associated proteins 1-5
BCRP: Breast cancer resistance protein
HTS: High-throughput screening
FDA: Food and Drug Administration
ELF4: E74-like ETS transcription factor 4
NEK8: NIMA-related kinase 8
TAK1: Transforming growth factor-*β*-activated kinase 1
TAB1: TGF-beta-activated kinase 1-binding protein 1
DUSP4: Dual-specific phosphatase 4
BITC: Benzyl isothiocyanate
LPS: Lipopolysaccharide
TLR: Toll-like receptor
CCE: Cinnamomi cortex extract
TRAIL: TNF-related apoptosis-inducing ligand
CRC: Colorectal cancer
ATO: Arsenic trioxide
HCC: Hepatocellular carcinoma
HNC: Head and neck cancer
TNBC: Triple negative breast cancer
PUMA: p53-upregulated modulator of apoptosis
PARP: Poly ADP ribose polymerase
CML: Chronic myeloid leukemia
NOD/SCID: Nonobese diabetic/severe combined immunodeficiency
EGCG: Epigallocatechin gallate
OSCC: Oral squamous cell carcinoma
TKI: Tyrosine kinase inhibitors
DHEA: Dehydroepiandrosterone
EGFR: Epidermal growth factor receptor
RITA: Reactivation of p53 and induction of tumor cell apoptosis
NADPH: Nicotinamide adenine dinucleotide phosphate reduced
ESC: Endometrial serous carcinoma
GCLC: Glutathione cysteine ligase catalytic subunit
ATRA: All-trans retinoic acid
APL: Acute promyelocytic leukemia
ALDH1: Aldehyde dehydrogenase1
BTZ: Bortezomib
GBM: Glioblastoma multiforme
TXNRD1: Thioredoxin reductase 1
TUSC2: Tumor suppressor candidate 2
LKB1: Liver kinase B1
VPA: Valproic acid
SOD2: Superoxide dismutase 2
IDH1: Isocitrate dehydrogenase 1
TET1: Ten-eleven translocation methylcytosine dioxygenase 1
SIRT1: Silent mating-type information regulation 1.
